# *N*,*N*′-Diaryldihydrophenazines as a Sustainable and Cost-Effective Alternative to Precious Metal Complexes in the Photoredox-Catalyzed Alkylation of Aryl Alkyl Ketones

**DOI:** 10.3390/molecules28010221

**Published:** 2022-12-27

**Authors:** Dmitry A. Dulov, Alexey V. Bogdanov, Sergey G. Dorofeev, Tatiana V. Magdesieva

**Affiliations:** Department of Chemistry, Lomonosov Moscow State University, Leninskie Gory 1-3, 119991 Moscow, Russia

**Keywords:** diaryldihydraphenazines, photoredox catalysis, thermally activated delayed fluorescence, triplet state, ESR, quantum chemical calculations, silyl enol ethers

## Abstract

An inexpensive and highly efficient metal-free alternative to commonly used Ru- and Ir-based catalysts was proposed. It was shown that the new 2,7-di-*tert*-butyl-5,10-bis(4-trifluoromethylphenyl)-5,10-dihydrophenazine outcompeted the iridium phenylpyridyl complex in photoredox activity in the alkylation of silyl enol ethers yielding aryl alkyl ketones. The reaction occurred under visible light irradiation at room temperature and was also applicable to drug derivatives (ibuprofen and naproxen). In-depth photophysical, electrochemical, and quantum chemical studies showed that the aforementioned *N*,*N*-diaryldihydrophenazine exhibited enhanced properties that were essential for the photoredox catalysis (a long-lived triplet excited state, strong reducing ability, high stability of the radical cations formed in single-electron-transfer event, and chemical inertness of the catalyst with respect to reactants). Importantly, the substituted *N*,*N*′-diaryldihydrophenazines could be obtained directly from diaryl amines; a facile, easily handled and scaled-up one-pot synthetic procedure was elaborated.

## 1. Introduction

Implementation of photoredox catalysis in organic synthesis is currently one of the most rapidly expanding areas [1]. This approach, which provides high chemical reactivity under mild conditions, has been successfully used for the construction of various types of covalent bonds (C-C, C-N, C-O, C-S, etc.). In the majority of early works, metal complexes (most often iridium or ruthenium bipyridyl derivatives) were used as photocatalysts and demonstrated excellent efficiency (due to redox reversibility, strong absorption of visible light, and an MLCT-stabilized excited state) [2,3,4]. Currently, the focus is gradually shifting to the search for and application of metal-free organic photocatalysts that are cheap, recyclable, non-toxic, and compatible with sustainable chemistry requirements [5,6]. To address this challenge, a number of organic photocatalysts have been suggested and tested in a wide variety of important structure-forming reactions [7,8,9,10].

The majority of organic metal-free photocatalysts described in the literature are excited-state oxidants (such as acridinium and pyrylium salts, rhodamine, Eosin Y, perylene diimides, and some others). Strongly reducing organic photocatalysts are more rare; among them, phenoxazines, phenothiazines, and dihydrophenazines should be mentioned (see [11,12,13] for reviews).

Taking into account the sustained need for abundant and reasonably priced metal-free photocatalysts, we turned our attention to *N*,*N*′-diaryldihydrophenazines. These compounds are easily accessible, and their skeleton provides wide possibilities for modification with various substituents. Moreover, the electronic properties of the phenazine core and those of the orthogonal *N*-aryl moieties can be independently tuned, thereby giving rise to the intramolecular charge-transfer excited state, which is reminiscent of the metal-to-ligand charge transfer (MLCT) in metal-containing catalysts. The reducing power of *N*,*N*′-diaryldihydrophenazines, which can form stable radical cations [14], is close to that of “classical” catalysts such as [Ru(bpy)_3_]^2+^ or [Ir(ppy)_3_] [15]. The compounds are sufficiently chemically inert and compatible with a wide range of catalytic processes. These features allow the consideration of dihydrophenazines as possible substitutes for expensive precious-metal-containing photocatalysts.

Meanwhile, the number of dihydrophenazines tested in photocatalytic applications is currently rather limited. Their structural diversity is mainly focused on variations in the *N*-aryl fragment [15,16,17,18]. The effect of dihydrophenazine core modification is less investigated [19]; the examples are limited to the conjugation-extending naphthyl moiety or phenyl groups.

Previous studies on the photocatalytic activity of dehydrophenazines were mainly focused on the atom-transfer radical polymerization driven by visible light [15,18,19,20,21] and provided promising results. Among other transformations, the decarboxylative olefination of activated aliphatic acids [16], the trifluoromethylation of olefins, as well as the C-*N*- and C-S cross-coupling of arylbromides with aliphatic amines and thioles [15] should be mentioned. These publications clearly demonstrated the great potential of *N*,*N*′-diaryldihydrophenazines as reducing photocatalysts. This highlights the challenges of expanding the range of available catalysts and transformations as well as further developing key structure/property relationships to determine the photocatalysts’ efficiency.

Herein, with the aim to provide new inexpensive visible-light photocatalysts, two new *N*,*N*′-diaryldihydrophenazines containing different substituents (*t*-Bu and CF_3_) in the *N*-aryl moieties and in the phenazine core, will be considered. A new, practical, and cost-efficient synthetic procedure will be elaborated that made the compounds easily available.

In-depth photophysical, electrochemical, and quantum chemical studies of the new compounds were performed that revealed their important features such as a long-living triplet state (which was directly observed using continuous-wave ESR spectroscopy), a strong reducing ability of the excited state, and delayed fluorescence (the TADF effect). When tested in the visible-light-induced alkylation of silyl enol ethers, the new dihydrophenazines showed much better catalytic performances than tris-2-phenylpyridinatoiridium(III) complex previously used in this synthetically important reaction [22]. Comprehensive analysis of the critical experimental and DFT-calculated parameters that determined the catalytic activity of the new substituted *N*,*N*′-diaryldihydrophenazines and of the Ir complex will be presented and discussed in the context of the “structure-property” relationships.

## 2. Results and Discussion

### 2.1. Synthesis and Characterization

**Scheme 1:**
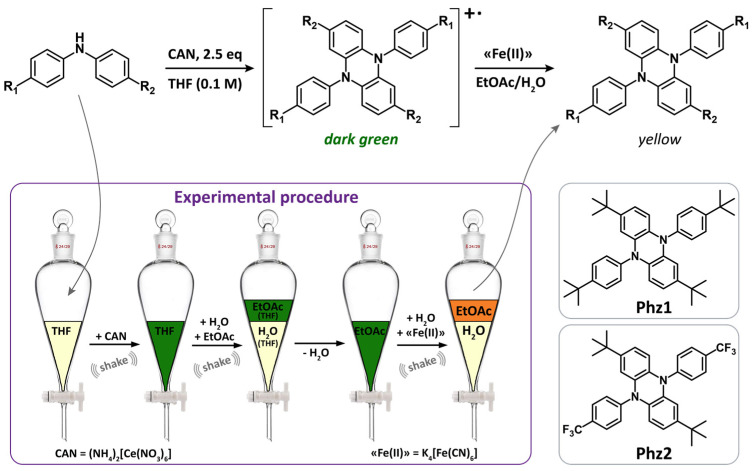
One-pot synthesis of dihydrophenazines **Phz1** and **Phz2**.

Two new *N*,*N*′-diaryldihydrophenazines were synthesized and fully characterized using spectral methods (see the Materials and Methods section). The synthetic one-pot procedure was very simple (Scheme 1) and showed a significant advantage of the new compounds over other organic photocatalysts. The synthesis was performed in a separatory funnel using ceric ammonium nitrate as an oxidant of the starting diarylamines to yield the corresponding radical cations. To convert the radical cationic species into the neutral *N*,*N*′-diaryldihydrophenazine, K_4_[Fe(CN)_6_] was added to the crude reaction mixture; then, after extraction with ethyl acetate, the targeted *N*,*N*′-diaryldihydrophenazines were isolated in a practical yield. It is worth mentioning that modification of the dihydrophenazine scaffold in previously reported photocatalysts was performed using Buchwald–Hartwig or Suzuki cross-coupling reactions [15,18,23]. Compared to this, the synthetic approach proposed in the present work was much more attractive due to the simplicity of the procedure as well as the affordability and low cost of the reagents.

The photophysical and electrochemical properties of the substituted *N*,*N*′-diaryldihydrophenazines were investigated.

### 2.2. Absorption and Emission Spectra

**Figure 1:**
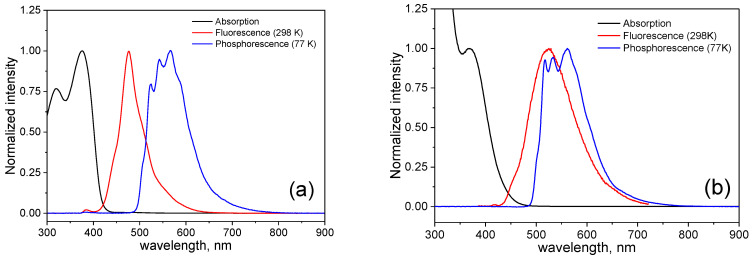
UV-vis absorption (black), emission (red) spectra (in benzene), and phosphorescence (blue) spectra (toluene, 77 K) for **Phz1** (**a**) and **Phz2** (**b**).

The UV-vis absorption and emission spectra for **Phz1** and **Phz2** are given in Figure 1.

The λ_max_ for the absorption peaks were close (376 nm for **Phz1** and 370 for **Phz2**); these were similar to the value (369 nm) for the S_0_→S_n_ excitation predicted for the unsubstituted *N*,*N*-diphenyldihydrophenazine [17]. The molar absorptivity ε was greater for **Phz1** (4499 vs. 3800 for **Phz2** in benzene), thereby making the S_0_→S_n_ transition more probable.

**Figure 2:**
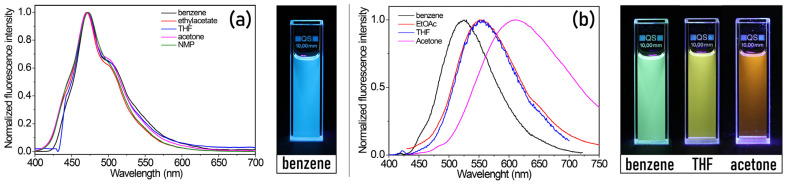
The emission spectra of **Phz1** (**a**) and **Phz2** (**b**) in various solvents.

The emission spectra of the compounds were significantly different. The emission band for **Phz2** was broader, redshifted (λ_max_ was 476 nm and 523 nm for **Phz1** and **Phz2**, respectively), and exhibited a much higher Stokes shift (177 nm for **Phz2** and 99 nm for **Phz1**), which indicated that the origin of the emission was based on the intramolecular charge transfer between the donor and acceptor units. A much higher Stokes shift for **Phz2** was attributed to a relaxation of the excited electron resulting in the LUMO having a lower energy due to the electron-withdrawing CF_3_ substituents, which was in contrast to the t-Bu groups in **Phz1** increasing the LUMO level. The emission spectrum for **Phz1** was insensitive to the solvent polarity, whereas a significant solvatochromic effect was observed for the emission of **Phz2** (Figure 2). The pronounced solvatochromism in the case of **Phz2** indicated emission from the polar charge-transfer state that is stabilized in polar solvents, thereby causing the redshift of the emission band. Thus, the nonpolar locally excited S1(LE) state was inherent to **Phz1**, which was in contrast to **Phz2**, for which the polar charge-transfer S1(CT) excited state is formed.

The lowest singlet excited-state energy values (E_S1_) were determined from the spectral data as the 0–0 transition energy calculated from the absorption and fluorescence onset wavelengths (2.96 eV for **Phz1** and 2.85 eV for **Phz2**). For the triplet states, the E_T1_ values were obtained similarly by using the absorption and phosphorescence onset wavelengths (2.74 eV for **Phz1** and 2.68 eV for **Phz2**).

### 2.3. ESR Data

**Figure 3:**
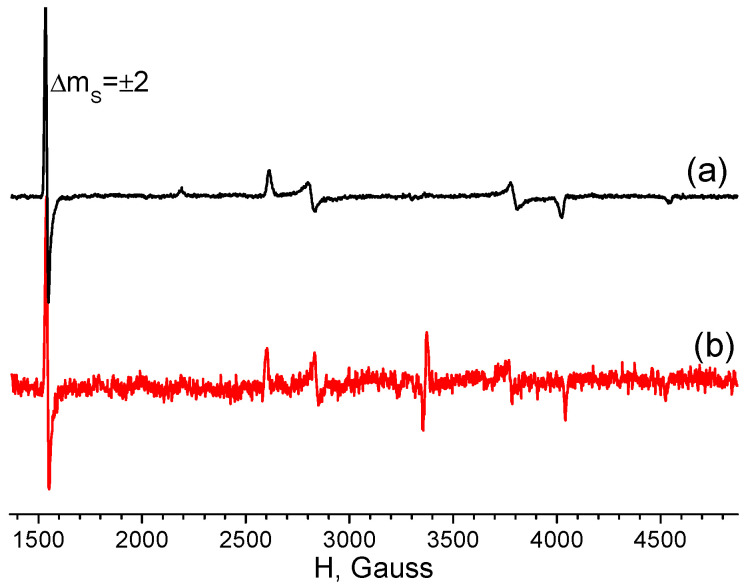
The ESR spectra recorded during continuous irradiation of toluene solutions of **Phz1** (**a**) and **Phz2** (**b**). The background spectra recorded for the same samples in the absence of light irradiation were subtracted.

The short-lived S1 state is often useless for photocatalysis; therefore, the ability to access the triplet state is important. Both the experimental and computation data revealed that the initial photon absorption and relaxation to the lowest excited S1 state (according to Kasha’s rule) was followed by the intersystem crossing to the lowest triplet state for both **Phz1** and **Phz2**. The direct observation of the triplet states of both dihydrophenazines was performed using ESR spectroscopy. The ESR spectra recorded at continuous irradiation of the supercooled toluene solutions of **Phz1** and **Phz2** inside the resonator of the ESR spectrometer are presented in Figure 3. Note that to reliably obtain the spectra of the photoinduced triplet state of the dihydrophenazines, the background spectra of the same samples in the absence of light were subtracted from the ESR spectra recorded under light irradiation (Figure S1).

The shapes of the spectra were typical for the ESR spectra of triplet molecules. The simulation of the ESR spectra is presented in Figure S2. The parameters of the zero-field splitting (determined by means of spectra simulations) were D = 1175G and E/D = 0.064, which are typical for photoexcited triplet molecules [24,25,26]. The lifetimes of the triplet states at 100 K determined via time-resolved ESR measurements were (1.26 ± 0.07) s and (0.97 ± 0.10) s for **Phz1** and **Phz2**, respectively (Figure S3).

### 2.4. Phosphorescence Spectra

**Figure 4:**
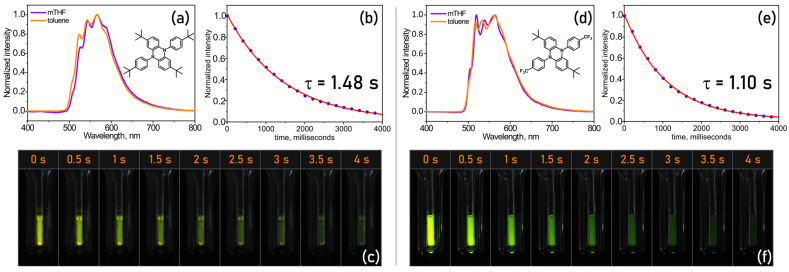
The phosphorescence spectra of **Phz1** (**a**) and **Phz2** (**d**) in various solvents (2-methyl-THF or toluene matrix at 77 K) and the phosphorescence lifetimes in the 2-methyl-THF matrix (**b**,**e**). Phosphorescence observed for **Phz1** (**c**) and **Phz2** (**f**) after the excitation source was turned off.

The phosphorescence spectra of the dihydrophenazines recorded at a low temperature were redshifted with respect to the emission spectra recorded at room temperature (Figure 1), which was evidence of the intersystem crossing of the excited molecules and the subsequent phosphorescence. The phosphorescence spectra for both **Phz1** and **Phz2** were almost independent of the solvent polarity (Figure 4), which indicated the nonpolar character of the triplet states T1(LE). It is worth noting that the phosphorescence of the frozen solutions of the dihydrophenazines could be visually observed (see Figure 4 below). The phosphorescence lifetimes determined via optical measurements were in considerable agreement with the ESR data (PhMe matrix: 1.50 s for **Phz1** and 1.0 s for **Phz2**; mTHF matrix: 1.48 s for **Phz1** and 1.1 s for **Phz2**).

**Figure 5:**
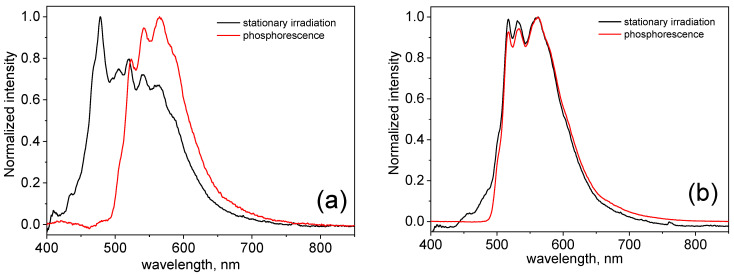
Low-temperature luminescence spectra for **Phz1** (**a**) and **Phz2** (**b**) under stationary light irradiation (black lines) and after turning off the excitation source (red lines).

Note that the low-temperature luminescence spectra for **Phz1** were different when they were recorded under stationary light irradiation (Figure 5, black lines) and when the luminescence was measured after the excitation source was turned off (Figure 5, red lines); **Phz2** demonstrated no such difference. This meant that the fluorescence lifetime for **Phz1** was of the same order of magnitude as the characteristic time of the intersystem crossing. Since these luminescence spectra comprised both fluorescence and phosphorescence, the S1-T1 intersystem crossing proceeded faster than fluorescence in the case of **Phz2**; therefore, the low-temperature luminescence was almost purely phosphorescence. This agreed well with the lower difference between the fluorescence and phosphorescence maxima observed for **Phz2** (Figure 1) because the small energy gap between the singlet and triplet excited states favored faster intersystem crossing for this molecule.

**Figure 6:**
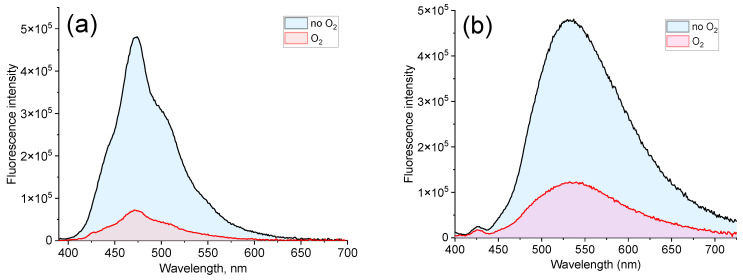
Luminescence spectra of toluene solutions of **Phz1** (**a**) and **Phz2** (**b**) deaerated prior to the measurement (black line) and bubbled with dioxygen (red line).

Another important photophysical observation related to photoluminescence of the dihydrophenazines under study was the significant quenching of the luminescence by dioxygen as illustrated in Figure 6. It can be seen that in both cases the shapes of the spectra remained the same, but the intensity of the luminescence was decreased in the presence of dioxygen. This observation suggested that at room temperature, the luminescence came not only from conventional prompt fluorescence, which would be insensitive to the presence of dioxygen, but also from delayed fluorescence. That meant that the part of the excited molecules underwent intersystem crossing to the triplet state, from which the radiative relaxation proceeded via the return to the singlet excited state and the fluorescence thereof. In the presence of dioxygen, some part of the long-living triplet molecules underwent radiationless relaxation to the ground state, thus quenching the luminescence.

The luminescence intensity was proportional to the intensity of the excitation light (Figure S10), thus suggesting that the observed luminescence corresponded to thermally activated delayed fluorescence (TADF) or E-type delayed fluorescence rather than to the P-type delayed fluorescence due to the triplet–triplet annihilation.

### 2.5. Frontier Orbital Localization

**Figure 7:**
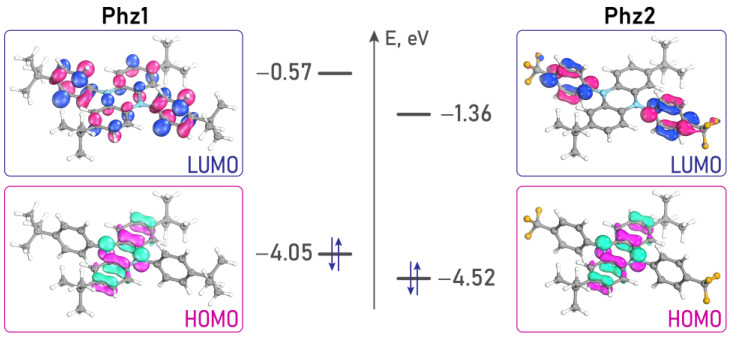
The HOMO/LUMO localization and the corresponding orbital energies for **Phz1** and **Phz2** (PBEh-3c).

Further insight into the photophysical properties of the dihydrophenazines was obtained by means of quantum chemical calculations. The HOMO and LUMO for the **Phz1** and **Phz2**, as well as the SOMOs of the oxidized state (see SI), were determined using the DFT approach (PBEh-3c). It can be seen in Figure 7 that the HOMO was localized at the phenazine core for both dihydrophenazines, whereas their LUMOs were different. First, the phenazine core had no impact on the LUMO of **Phz2**, which was delocalized exclusively over the *N*-aryl moieties. In contrast, the LUMO of **Phz1** had a certain impact on the phenazine core, although the contribution of the *N*-phenyl moieties was dominant. Second, the orbitals of the *N*-phenyl rings that contributed to the LUMOs of **Phz1** and **Phz2** were symmetrically different (although both had two nodal planes).

Thus, **Phz2** had spatially disjoint frontier orbitals, which suggested an occurrence of an intramolecular charge transfer in the lowest excited state. As for **Phz1**, the overlap between the modulus of the HOMO and LUMO could be detected. A small overlap between the frontier orbitals led to a weak exchange interaction, thus minimizing the exchange integral (J). The value of the singlet–triplet energy gap (ΔE_ST_) in turn was equal to the doubled exchange integral [27].

The calculation of the J values for **Phz1** and **Phz2** allowed us to estimate the ΔE_ST_ values for **Phz1** (0.22 ± 0.04 eV) and **Phz2** (0.01 eV; see Table S3). As could be expected for the HOMO/LUMO localizations given in Figure 7, the intersystem crossing in **Phz2** was more favorable. However, the ΔE_ST_ value obtained for **Phz1** was not high enough to prevent the triplet state formation. Indeed, the triplet states were detected for both compounds (see above). Thus, the DFT estimation was in agreement with the experimental results (Table 1). The lower ΔE_ST_ value determined for **Phz2** was in agreement with the transition between the S1(CT) and T1(LE) states, which were closer in energy [28] than the S1(LE) and T1(LE) states inherent to **Phz1**.

Notably, the small singlet–triplet energy gap (ΔE_ST_ < 0.3 eV) is an important precondition for reverse intersystem crossing at ambient temperatures that gives rise to the delayed fluorescence phenomenon (TADF) [30]. The efficient upconversion of triplet excitons into a singlet state opens a pathway for obtaining high-efficiency luminescence. Reverse intersystem crossing can be realized by harvesting environmental thermal energy. Spatially separated and electronically decoupled donor–acceptor compounds with large torsion angles between the donor and acceptor fragments are known to be the best candidates for TADF [28,31,32]. **Phz1** and **Phz2** matched these requirements and may be of practical interest.

### 2.6. Redox Behavior

**Figure 8:**
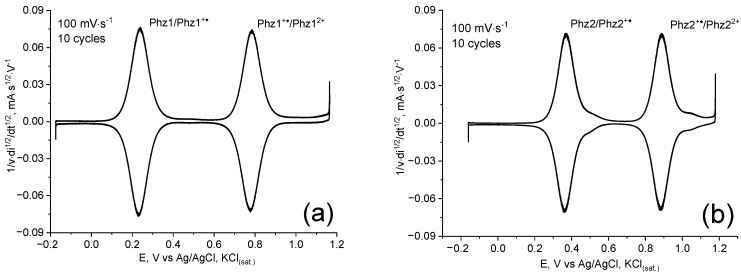
Semi-differential voltammograms obtained for **Phz1** (**a**) and **Phz2** (**b**) upon multiple cycles in a −0.2 V–1.2 V potential range (Pt, THF, 0.2 M Bu_4_NBF_4_ 100 mV/s, 10 cycles).

The reducing ability of the catalyst and the stability of the oxidized state are critical features that determine the possibility of its application in photoredox catalysis. The oxidation potential values for **Phz1** and **Phz2** were determined using cyclic voltammetry with a Pt disc electrode in a THF solution. Both compounds were easily oxidized; in both cases, two completely reversible redox couples were detected (Figure 8) that corresponded to the formation of the stable radical cation and dicationic species. The voltammetric patterns remained unchanged during multiple cycles in a −0.2 V–1.2 V potential range, which indicated a high stability of the oxidized states. The oxidation potential values for **Phz2** were anodically shifted relative to **Phz1** due to electron-withdrawing CF_3_ groups in the *N*-aryl fragments, whereas the potential gaps between two consecutive oxidations were similar for both compounds.

The reducing ability of the photoexcited state of the dihydrophenazines could be estimated by combining the spectral and electrochemical data. The experimental singlet excited-state reduction potential values were estimated using the following Equation [27]:


$$E_{{PC}^{+}/{PC}^{*}}^{*0}=E_{{PC}^{+}/PC}^{0}-\Delta G^{0}(\mathrm{excited}\;\mathrm{state}-\mathrm{ground}\;\mathrm{state})$$

The values obtained for both *N*,*N*′-diaryldihydrophenazines did not differ significantly (see Table 1), although the excited state of **Phz1** had a higher reducing ability. Generally, the excited state potentials (E_Ox_*) were responsible for the initiation of the photocatalytic cycle, whereas the E_Ox_ values were essential to close the cycle, thereby restoring the ground state of the photocatalyst. The E_Ox_ values of **Phz1** and **Phz2** were well-correlated with the HOMO levels.

The most important characteristics of **Phz1** and **Phz2** that will be essential to their functioning as photoredox catalysts are summarized in Table 1. For comparison, the corresponding parameters for the previously reported *N*,*N*′-dinaphtyldihydrophenazine (NaphtPhz) (which showed a high photocatalytic activity in the trifluoromethylation of alkenes) [15] and Ir(ppy)_3_ complex [29] are also presented. The comparative analysis is given below in the context of the catalytic testing results.

### 2.7. Testing in Photoredox-Catalyzed Decarboxylative Alkylation of Silyl Enol Ethers

**Scheme 2:**
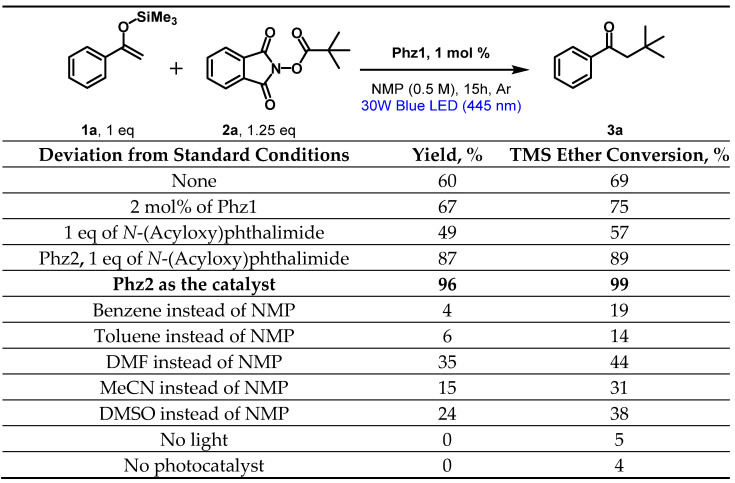
Optimization of the reaction conditions.

The analysis of the intrinsic photophysical properties given above was used to correlate the catalytic performance of the new dihydrophenazines with their structures. As a model reaction, the alkylation of silyl enol ethers was chosen because it provides a direct route to variously functionalized ketones that are important building blocks for the synthesis of biologically active compounds and other practically important molecules. One of the possible routes to these types of compounds is the photoredox-catalyzed decarboxylation of *N*-(acyloxy)phthalimide, which can serve as an alkyl radical source [33,34,35] (Scheme 2). In a recently reported paper [22], a number of ruthenium and iridium complexes containing bipyridyl and phenylpyridine ligands were tested as photocatalysts to promote the α-alkylation of acetophenone using a wide range of *N*-(acyloxy)phthalimides. Replacing the expensive precious-metal-containing catalysts with cheaper ones without losing efficiency is very tempting. This prompted us to try dihydrophenazines as catalysts in this reaction. Two tasks were set: to compare the photocatalytic activities of **Phz1** and **Phz2** in the context of the results of their photophysical and electrochemical studies as well as to elaborate a facile, practical, and precious-metal-free reaction protocol to yield α-alkylated arylalkyl ketones.

The optimization of the reaction conditions was started by using **Phz1** (1 mol%) as the catalyst (Scheme 2). Silyl enol ether and *N*-pivaloylphthalimide (in the equimolar ratio) were taken as the reactants. The screening of the solvents indicated that NMP provided a much better result than the other polar solvents; the aromatic solvents (benzene, toluene) were not suitable at all. An increase in the amount of the catalyst (2 mol%) enhanced the yield of the targeted product, while a significant amount of the starting enol was detected in the reaction mixture. The testing of **Phz2** in the same reaction conditions provided much better results (88% yield of the targeted product and 8% of the unreacted enol). Notably, it turned out that even 1 mol% of **Phz2** was enough to obtain an 87% yield of phenyl-*neo*-pentylketone. A 25% excess of *N*-pivaloylphthalimide in the experiments with 1 mol% of **Phz2** allowed us to obtain phenyl-*neo*-pentylketone in an almost quantitative yield. The control experiments revealed that visible light irradiation and the catalyst were essential for the reaction to occur.

**Scheme 3:**
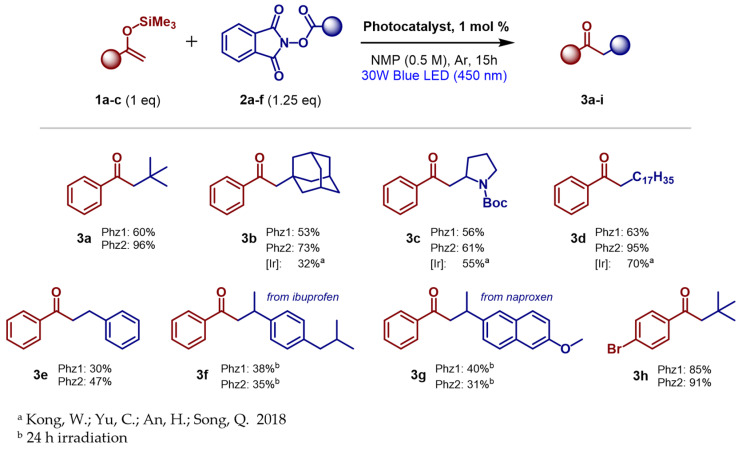
Photoredox-catalyzed decarboxylative alkylation of silyl enol ethers [22].

With the optimized reaction conditions in hand, other *N*-(acyloxy)phthalimides were tested in the reaction. For comparison of the catalytic activities of our dihydrophenazines with those of the iridium complexes_,_ three challenging substrates were chosen. These were derivatives of the sterically demanding adamantyl carboxylic acid, that of the *N*-protected α-amino acid (l-proline) important for drug design, and *N*-(acyloxy)phthalimide derived from stearic acid. These compounds were also used in [22] as the substrates in the same reaction but with the different photocatalysts (Ir(ppy)(dtbbpy)_2_](PF_6_) or Ir(ppy)_3_). In addition to the aforementioned compounds, the derivatives of pivalic and phenyl acetic acids were tested. Decarboxylation of these acids provided stabilized tert-butyl and benzyl radicals; therefore, a low reactivity could be expected when taking into account the results obtained in [22]. However, the *N*-(acyloxy)phthalimide of pivalic acid provided almost quantitative yields of the ketones derived from both acetophenone and 4-bromoacetophenone when **Phz2** was used as the photocatalyst. In the case of the benzyl radical, the yield was not that high (47% only) but it significantly exceeds that obtained in [22]. The results are summarized in Scheme 3. Thus, primary, secondary, and even more sterically demanding and less active tertiary alkyl radicals could be inserted in the α-position to yield a variety of aryl alkyl ketones. **Phz2** showed a superior performance as compared to the iridium counterparts; even **Phz1,** which was inferior in activity to **Phz2**, provided better results than the iridium complex in some cases.

The performances of drug molecules such as ibuprofen and naproxen were also tested. An innovative approach to drug design based on a multitarget strategy requires the development of covalent combinations of lipophilic nonsteroidal anti-inflammatory drugs (e.g., ibuprofen) or other pharmaceuticals with various molecular transporters, thus improving cell penetration [36,37].To address this challenge, the development of convenient approaches to chemical grafting to yield drug conjugates is important. The testing of **Phz1** and **Phz2** in the photocatalyzed reaction of the drug derivatives resulted in a ca. 40% yield of the corresponding ketones.

To shed light on the observed difference in the photocatalytic activities of **Phz1** and **Phz2** and to establish a “structure-property” relationship, the photophysical and electrochemical parameters were compared. Both compounds absorbed the blue light (450 nm) used for irradiation, but the molar extinction at this wavelength was higher for **Phz2**. The lowest-energy excited states of **Phz1** and **Phz2** formed upon irradiation were of a different character: S1(LE) for the former and S1(CT) for the latter. The intersystem crossing to the lowest triplet state was possible for both dihydrophenazines due to relatively low ΔE_ST_ values, although the S1-T1 transition was more favorable for **Phz2**, which had a smaller ΔE_ST_ value. It was reasonable to assume that the concentration of molecules in the triplet state would be higher for **Phz2**.

Both compounds exhibited the TADF effect. For TADF compounds with a small ΔE_ST_ value, the rate of the electron transfer from the S1 or T1 excited state of the catalyst to the substrate should not be significantly different, and both states can be productive in photoredox catalysis [38]. However, the triplet state has an advantage of a longer excited state lifetime, thereby making the electron transfer more competitive to a radiative decay. The exact populations of the singlet and triplet states are determined by the respective quantum yields and lifetimes of the prompt and delayed fluorescence. In the context of this logic, the higher photocatalytic efficiency observed for **Phz2** in the catalysis can be mainly attributed to the higher lifetime of the triplet state (see Table 1). In a previous study [21], it was also implicated that the triplet state of the TADF dihydrophenazine catalyst was responsible for the atom-transfer radical polymerization.

Notably, although the **Phz2** that exhibited a strong intramolecular charge transfer (due to the substituents of the opposite electronic nature in the phenazine core and in the *N*-aryl fragments) showed a superior catalytic efficiency, **Phz1** that formed a locally excited singlet state also exhibited significant activity that resulted in practical yields of aryl alkyl ketones. It seemed that the *N*,*N*′-diaryldihydrophenazines geometry itself already facilitated the HOMO-LUMO spatial separation, even in the case when both the dihydrophenazine core and the *N*-aryl moiety had donor substituents and *a priori* it was not obvious which part of the molecule was a donor and which was an acceptor. On the other hand, the electron-donating substituents facilitated the ground-state oxidation, thereby increasing the reducing power of the excited state, which may have been beneficial for the catalysis. A comparison with the previously reported NaphtPhz [15] showed that its benefit was a higher extinction coefficient due to the polyaromatic fragments. However, it lost to **Phz2** in other parameters such as the lifetime and the yield of the triplet state, as well as in the reducing ability of the excited state. Thus, even subtle *N*,*N*′-diaryldihydrophenazine scaffold-based variations (which can be easily accomplished using known synthetic protocols) offer wide possibilities to develop a tailored photocatalyst for each specific reaction.

As compared to the Ir(ppy)_3_ complex, **Phz2** had a much longer excited state lifetime (Table 1). Moreover, the reducing abilities of **Phz1** and **Phz2** were significantly higher compared to those of the Ir catalysts. These key differences were contributing factors to the success of the new dihydrophenazines in replacing precious metal complexes in photoredox catalysis; this was proved using the photoredox-catalyzed decarboxylative alkylation of silyl enol ethers as a model process.

## 3. Conclusions

Two differently substituted *N*,*N*′-diaryldihydrophenazines were obtained in high yields directly from the substituted diaryl amines using an inexpensive, easily handled, and scaled-up one-pot procedure. Extended photophysical, electrochemical, and quantum chemical studies of the new *N*,*N*′-diaryldihydrophenazines showed that the compounds exhibited enhanced photocatalytic properties (such as a long-lived triplet excited state, a stronger reducing ability, a high stability of the radical cations formed in single-electron-transfer event, and chemical inertness of the catalyst with respect to the reactants). The direct observation of the triplet states of the dihydrophenazines was performed using continuous-wave ESR spectroscopy. The 2,7-di-*tert*-butyl-5,10-bis(4-trifluoromethylphenyl)-5,10-dihydrophenazine (**Phz2**) outcompeted the precious metal counterparts in the alkylation of silyl enol ethers to yield aryl alkyl ketones.

The sustained need for abundant, reasonably priced, and metal-free photocatalysts make these new compounds highly attractive alternatives to the commonly used Ru- and Ir-based catalysts.

## 4. Materials and Methods

### 4.1. General Information

The solvents and reagents were obtained from commercial sources (Merck, Darmstadt, Germany) and used without purification unless otherwise stated. The *N*-methyl-2-pyrrolidone (NMP) and dimethylsulfoxide were distilled over CaH_2_ under Ar. The MeCN and DMF were distilled over P_2_O_5_ under Ar. The benzene, toluene, and THF were distilled over Na/benzophenone under Ar. The hexane, DCM, and EtOAc were distilled over CaCl_2_. For the chromatographic purification of the reaction products, silica gel (40–63 µm, Macherey-Nagel, Düren, Germany) was used. Argon (99.995%) was used as the inert gas source for the glovebox.

The NMR spectra (^1^H, ^13^C{^1^H}, ^19^F) were recorded using an Agilent 400-MR (Agilent, Santa Clara, CA, USA); the chemical shifts were corrected relative to chemical shift of a non-deuterated solvent. The HRMS spectra were recorded using an AB Sciex TripleTOF 5600+ instrument with a TurboV ion source with an APCI probe installed and a photospray ion source (SCIEX, Framingham, MA, USA). The GC/MS analyses of the reaction mixtures were performed using an Agilent 8890/5977B system (Agilent, Santa Clara, CA, USA). The GC/FID analyses were performed using a Chromatec Crystal 5000.2 device (Chromatec, Yoshkar-Ola, Russia). The absorption spectra were recorded for the benzene solutions using an AvaSpec-2048 instrument (Avantes B.V., Apeldoorn, Netherlands). The fluorescence measurements were made using a Horiba Fluoromax instrument (HORIBA, Irvine, CA, USA); the fluorescence quantum yields were determined using a Rhodamine 6G as a standard. The Electrochemical measurements were carried out using 2 mM THF solutions in a three-electrode electrochemical cell (Pt working and counter electrodes; Ag/Ag^+^, 0.1 M Bu_4_NBF_4_-MeCN reference electrode) using a Biologic BP-300 instrument (Biologic, Seyssinet-Pariset, France). The EPR spectra were recorded using a Bruker EMX plus X-band spectrometer (Bruker, Billerica, MA, USA). The temperature of the samples was controlled using a flow of nitrogen gas. The spectra were recorded at (100 ± 0.5) K; for more details, see the Supplementary Materials.

The TMS enol ethers and *N*-acyloxyphthalimides were obtained as previously reported (see the Supplementary Materials for the details).

### 4.2. Computational Details

A stationary-point structure search was performed using the ORCA 5.0.3 quantum chemistry package (Max-Planck-Institut für Kohlenforschung, Mülheim an der Ruhr, Germany) [39]. The PBEh-3c composite method [40] was applied for the geometry optimizations and analytical Hessian calculations. In the optimization procedures, a threshold of 1 × 10^−9^ Hartree was used for the SCF convergence and thresholds of 1·10^−6^ Hartree and 3 × 10^−5^ Hartree·Bohr^−1^ for the energy and RMS gradients, respectively, were employed. Hessian calculations were carried out to confirm that the structures found were the actual minima or saddle points. The Multiwfn program (Beijing Kein Research Center for Natural Sciences, Beijing, China) [41] was used to calculate the exchange integrals between the HOMO and LUMO.

### 4.3. Synthesis of Diaryldihydrophenazines

A THF solution of diarylamine (1 eq., 0.1 M) was placed in a separatory funnel. A finely ground powder of ceric ammonium nitrate (2.5 eq.) was added; the mixture was then intensively shaken for 30 s and equal volumes of water and ethylacetate were added. After shaking, the lower (colorless) layer was removed; the resulting solution was then washed with water. An aqueous solution of potassium ferrocyanide (2 eq., 0.1 M) was added, and the resulting mixture was shaken until the dark-green color disappeared. The brownish-orange organic layer was dried over anhydrous Na_2_SO_4_, and the solvent was removed under reduced pressure. The residue was recrystallized from benzene to yield the desired product as a solid powder.

*2,7-di-tert-butyl-5,10-bis(4-(tert-butyl)phenyl)-5,10-dihydrophenazine (Phz1)*

**Phz1** was obtained from 4,4‘-di-*tert*-butyldiphenylamine in an 80% yield as an off-white powder.

**^1^H NMR** (C_6_D_6_): δ 7.37–7.31 (m, 4H), 6.33 (dd, *J* = 8.1, *J* = 1.8 Hz, 1H), 6.14 (d, *J* = 1.7 Hz, 1H), 5.94 (d, *J* = 8.2 Hz, 1H), 1.20 (s, 9H), 1.07 (s, 9H). **^13^C NMR** (C_6_D_6_) δ 150.90, 143.80, 138.74, 137.12, 135.22, 131.19, 127.85 (detected by HSQC; overlap with solvent signal), 117.48, 113.01, 110.65, 34.69, 33.96, 31.41, 31.29. **HRMS (APCI-TOF):** *m*/*z* = 559.4043 ([M + H]^+^, 559.4047 calculated for C_40_H_51_N_2_^+^) **MS (EI)** *m*/*z* (%): 559.4 (44), **558.4 (100, M)**, 543.4 (10), 279.2 (25), 271.6 (18), 264.1 (39), 250.1 (25).

*2,7-di-tert-butyl-5,10-bis(4-trifluoromethylphenyl)-5,10-dihydrophenazine (Phz2)*

**Phz2** was obtained as a yellow powder in a 40% yield from 4-*tert*-butyl-4′-trifluoromethyldiphenylamine. To suppress the deprotonation of the radical cation of the starting amine, 1 eq. of HCl (conc.) was added to a THF solution prior to the CAN addition.

**^1^H NMR** (THF-d8): δ 7.94–7.90 (m, 4H), 7.61–7.55 (m, 4H), 6.52 (dd, ^3^*J* = 8.3, ^4^*J* = 2.1 Hz, 2H), 6.06 (d, ^4^*J* = 2.1 Hz, 2H), 5.92 (d, ^3^*J* = 8.3 Hz, 2H), 1.05 (s, 18H). **^13^C NMR** (THF-d8): δ 146.66, 145.75, 136.97, 135.07, 131.02, 129.90 (q, ^2^*J*_C-F_ = 32.4 Hz), 129.00 (q, ^3^*J*_C-F_ = 3.6 Hz), 125.46 (q, ^1^*J*_C-F_ = 271.8 Hz), 119.18, 115.58, 113.35, 34.73, 31.54. **^19^F NMR** (THF-d8): δ −59.45. **HRMS (ESI):** *m*/*z* = 583.2538 ([M + H]^+^, 583.2542 calculated for C_34_H_33_F_6_N_2_^+^) **MS (EI)** *m*/*z* (%): 583.3 (37), **582.3 (100, M)**, 567.3 (23), 437.2 (12), 407.2 (18), 391.1 (23), 377.1 (14), 351.1 (13), 291.1 (18), 283.6 (17), 276.1 (58), 262.1 (29), 248.1 (63), 145 (14).

### 4.4. Photoredox Alkylation of TMS Enol Ethers

In the glovebox, a 4 mL vial was charged with TMS enol ether **1** (0.25 mmol, 1 eq.), *N*-acyloxyphthalimide **2** (0.31 mmol, 1.25 eq), the photocatalyst (**Phz1** or **Phz2,** 2.5 × 10^−4^ mmol, 0.01 eq.), and 0.5 mL of dry NMP. The mixture was stirred for 5 min then sealed with screw cap with a septum and transferred into the photoreactor (30 W 450 nm LED). After 15 h of irradiation under stirring, the mixture was diluted with 7 mL of water and extracted with Et_2_O (3 × 4 mL). The combined organic fractions were washed with brine and dried over anhydrous Na_2_SO_4_, then the solvent was removed under reduced pressure. The residue was purified using column chromatography. For product characterizations, see the Supplementary Materials.

## Figures and Tables

**Table 1:** Key parameters that determined the photoredox catalytic activity of **Phz1** and **Phz2** as well as those for the Ir(ppy)_3_ complex and *N*,*N*′-dinaphtyldihydrophenazine (NaphtPhz).

	Phz1	Phz2	NaphtPhz [15]	Ir(ppy)_3_ [29]
λ_max_(abs), nm	375	370	343	377
ε(λ_max_)	4400	3800	5950	13100
τ(TADF, 298 K), μs	<10	65	4.3	2.1
E_ST_(exp), eV	0.22	0.11	-	~0
E_ST_(calc), eV	0.22 ± 0.04	0.01	-	-
τ(phos, 77 K, toluene)	1.5 sec	1.0 sec	-	~10 sec
Photoluminescence quantum yield _PL_, %	0.06	0.03	-	1
Quantum yield of triplet state formation, _T_, %	0.05	0.02	0.02	1
E(Ox), V vs. Ag/AgCl	0.24	0.37	0.25	0.81
E(Ox)*_T1_, V vs. Ag/AgCl	−2.5	−2.31	−1.65	−1.69

## Data Availability

Not applicable.

## Supplementary Materials

The following supporting information can be downloaded at: https://www.mdpi.com/article/10.3390/molecules28010221/s1, Experimental procedures, product characterization, and GMS and NMR spectra; Figure S1: ESR spectrum of Phz1 in toluene (100 K); Figure S2: Experimental and simulated spectrum of the photoexcited triplet state of Phz1 in toluene at 100 K; Figure S3: Time dependence of ESR signal for the triplet forms of Phz1 (a) and Phz2 (b) in toluene at 100 K; Figure S4: Ad hoc quartz EPR cell used in the experiments; Figure S5: Time dependence of fluorescence intensity for deaerated solutions of Phz1 and Phz2 in toluene; Figure S6: Experimental setup for the phosphorescence lifetime measurement; Figure S7: Experimental scheme for the phosphorescence lifetime measurement; Figure S8: Time dependence of integral phosphorescence intensity for glassified solutions of Phz1; Figure S9: Time dependence of integral phosphorescence intensity for glassified solutions of Phz2; Figure S10: Fluorescence intensity vs. the power of the excitation light source for the toluene solution of Phz1; Figure S11: A 3D model of the photoreactor; Figure S12: The photoreactor at work (left) and the complete experimental setup (right); Figure S13: Spectrum of the light source used in the photoreactor; Figure S14: Cyclic voltammograms for Phz1 (left) and Phz2 (right); Figure S15: Semi-differential (left) and semi-integral (right) voltammograms for Phz1; Figure S16: Semi-differential (left) and semi-integral (right) voltammograms for Phz2; Table S1: Photophysical parameters for Phz1 and Phz2 determined based on spectral data; Table S2: Electrochemical characteristics for Phz1 and Phz2; Table S3: ΔE_ST_ values for Phz1 and Phz2.
