# Supplementary material for: N,N′-Diaryldihydrophenazines as a Sustainable and Cost-Effective Alternative to Precious Metal Complexes in the Photoredox-Catalyzed Alkylation of Aryl Alkyl Ketones
Source: Molecules. 2022 Dec 27;28(1):221. doi: 10.3390/molecules28010221 (PMC9822323; doi:10.3390/molecules28010221)
Supplement: Supplementary file 1 [file molecules-28-00221-s001.zip › molecules-2097798-supplementary.pdf]

Contents

1. Synthetic procedures and substance characterizations 2
2. ESR measurements of photoexcited triplet states 6
3. Photophysical experiments 9
4. Experimental setup for photochemical experiments 13
5. Electrochemical measurments 15
7. HRMS spectra 22
8. NMR spectra 25

1. Synthetic procedures and substance characterizations

Synthesis of TMS enol ethers

In the glovebox, in a vial containing a mixture of ketone (1 equiv.), pre-dried sodium iodide (1.2 equiv.), and dry acetonitrile (1.5 mL/mmol), triethylamine (1.5 equiv.) was added followed by chlorotrimethylsilane (1.2 equiv.). The reaction mixture was stirred overnight at room temperature. The reaction was quenched with a mixture of hexane (50 mL) and water. The organic phase was separated and the aqueous layer was extracted with hexane (2 × 30 mL). The combined organic fractions were dried over anhydrous Na_2SO_4. The solvent was removed under reduced pressure and the residue was purified via distillation.

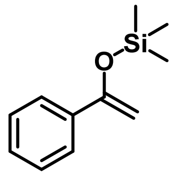

1a

*Trimethyl((1-phenylvinyl)oxy)silane (**1a**).* The 1a was obtained as a colorless liquid in a 79% yield.

^{1}**H NMR** ($CDCl_3$): δ 0.34 (9H, s), 4.50 (1H, d, J = 1.6 Hz), 4.99 (1H, d, J = 1.6 Hz), 7.33-7.40 (3H, m), 7.66-7.68 (3H, m).

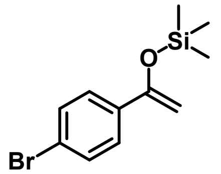

1b

*((1-(4-Bromophenyl)vinyl)oxy)trimethylsilane (**1b**).* The 1b was obtained as a colorless liquid in an 84% yield.

^{1}**H NMR** ($CDCl_3$): δ 7.47 – 7.42 (m, 4H), 4.90 (d, J = 1.9 Hz, 1H), 4.44 (d, J = 1.9 Hz, 1H), 0.27 (s, 9H).

Synthesis of N-(acyloxy)phthalimides **2** (general procedure)

To a stirred mixture of N-hydroxyphthalimide (1.0 eq.), carboxylic acid (1.2 eq.), and N,N-dimethylaminopyridine (0.05 eq.) in DCM (1 M), a solution of DCC in DCM (1.2 eq., 1 M) was added dropwise. The mixture was stirred for 24 h at room temperature and then filtered through a glass frit. The filtrate was evaporated, and the residue was purified using column chromatography.

*1,3-Dioxoisoindolin-2-yl pivalate (**2a**).* A two-necked flask was evacuated and filled with argon; N-hydroxyphthalimide (10.4 mmol) and a solution of pivaloyl chloride (9.5 mmol) in DCM (50 ml) were then added. Et_3N (10.4 mmol) was added dropwise under stirring. The resulting clear orange mixture was stored under argon for 2 days. Afterward, the solvent was removed under a vacuum, and the residue was dispersed in 40 ml of Et_2O. The resulting mixture was filtered, evaporated, and passed through a silica plug (d = 35 mm, h = 30 mm; eluent hexane/EtOAc 5:1). Evaporation of the resulted solution provided 2.23 g (95% yield) of **2a** as a white solid.

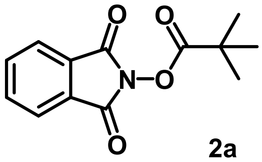

2a

^{1}**H NMR** ($CDCl_3$):δ 7.91 – 7.85 (m, 2H), 7.81 – 7.76 (m, 2H), 1.43 (s, 9H).

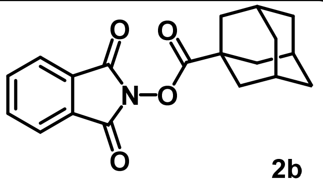

2b

*1,3-Dioxoisoindolin-2-yl adamantane-1-carboxylate (**2b**).* According to the general procedure, **2b** was obtained from adamantane-1-carboxylic acid as a white solid (74% yield, eluent Hex/DCM 1:1, R_f = 0.3).

^{1}H NMR ($CDCl_3$): δ 7.90 – 7.84 (m, 2H), 7.80 – 7.74 (m, 2H), 2.16 – 2.12 (m, 6H), 2.12 – 2.08 (m, 3H), 1.80 – 1.76 (m, 6H).

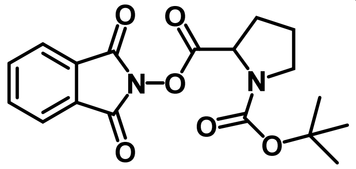

2c

*1-(Tert-butyl)-2-(1,3-dioxoisoindolin-2-yl)-pyrrolidine-1,2-dicarboxylate (**2c**).* According to the general procedure, **2c** was obtained from Boc-protected L-proline as a white solid (90% yield, eluent Hex/DCM 1:1, R_f = 0.3).

^{1}H NMR ($CDCl_3$, two rotamers): δ 7.90 – 7.84 (m, 2H), 7.81 – 7.76 (m, 2H), 4.72 – 4.57 (m, 1H), 3.66 – 3.57 (m, 1H), 3.52 – 3.43 (m, 1H), 2.48 – 2.30 (m, 2H), 2.11 – 1.94 (m, 2H), 1.52 – 1.46 (m, 9H).

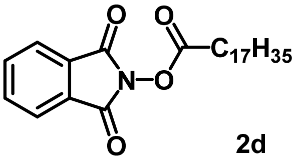

2d

*1,3-Dioxoisoindolin-2-yl stearate (**2d**).* According to the general procedure, **2d** was obtained from stearic acid as a white solid (85% yield, recryst. from hexane).

^{1}H NMR ($CDCl_3$): δ 7.91 – 7.86 (m, 2H), 7.81 – 7.76 (m, 2H), 2.66 (t, J = 7.5 Hz, 2H), 1.83 – 1.73 (m, 2H), 1.48 – 1.19 (m, 28H), 0.91 – 0.84 (m, 3H).

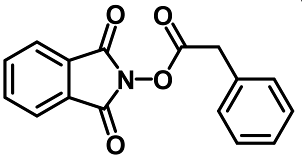

2e

*1,3-Dioxoisoindolin-2-yl 2-phenylacetate (**2e**).* According to the general procedure, **2e** was obtained from phenylacetic acid as a yellowish solid (82% yield, eluent Hex/DCM 1:1, R_f = 0.3).

^{1}H NMR ($CDCl_3$): δ 7.91 – 7.86 (m, 2H), 7.81 – 7.76 (m, 2H), 7.40 – 7.30 (m, 5H), 4.00 (s, 2H).

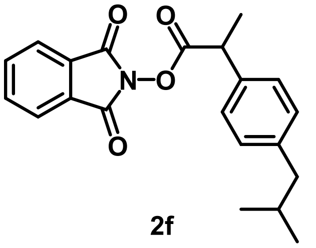

2f

*1,3-Dioxoisoindolin-2-yl 2-(4-isobutylphenyl)propanoate (**2f**).* According to the general procedure, **2f** was obtained from ibuprofen as a colorless oil (99% yield, eluent Hex/DCM 1:1, R_f = 0.3).

^{1}H NMR ($CDCl_3$): δ 7.89 – 7.83 (m, 2H), 7.80 – 7.75 (m, 2H), 7.34 – 7.28 (m, 2H), 7.19 – 7.14 (m, 2H), 4.10 (q, J = 7.2 Hz, 1H), 2.47 (d, J = 7.2 Hz, 2H), 1.87 (m, 1H), 1.66 (d, J = 7.2 Hz, 3H), 0.91 (d, J = 6.6 Hz, 6H).

^{13}C NMR ($CDCl_3$): δ 171.08, 161.99, 141.40, 135.71, 134.83, 129.74, 129.11, 127.39, 124.03, 45.21, 42.73, 30.28, 22.54, 19.16.

HRMS (APCI-TOF): m/z = 352.1542 ([M+H]+, 352.1543 calculated for $C_{21}H_{22}NO_4^+$).

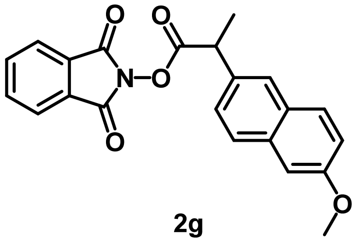

2g

*1,3-Dioxoisoindolin-2-yl (S)-2-(6-methoxynaphthalen-2-yl)propanoate (**2g**).* According to the general procedure, **2g** was obtained from naproxen as a white solid (70% yield, eluent Hex/DCM 1:1, R_f = 0.2).

^{1}**H NMR** ($CDCl_3$): δ 7.85 – 7.71 (m, 7H), 7.49 (dd, ^{3}J = 8.5, ^{4}J = 1.9 Hz, 1H), 7.19 – 7.12 (m, 2H), 4.27 (q, ^{3}J = 7.1 Hz, 1H), 3.91 (s, 3H), 1.75 (d, ^{3}J = 7.1 Hz, 3H).

^{13}**C NMR** ($CDCl_3$): δ 171.04, 161.98, 158.03, 134.82, 134.13, 133.58, 129.56, 129.08, 129.07, 127.67, 126.49, 126.03, 124.02, 119.31, 105.81, 55.46, 43.08, 19.15.

HRMS (APCI-TOF): m/z = 376.1180 ([M+H]+, 376.1179 calculated for $C_{22}H_{18}NO_5^+$).

Photoredox alkylation of TMS enol ethers

In the glovebox, a 4 ml vial was charged with TMS enol ether **1** (0.25 mmol, 1 eq.), N-acyloxy-phthalimide **2** (0.31 mmol, 1.25 eq.), the photocatalyst (**Phz1** or **Phz2,** $2.5 \cdot 10^{-4}$ mmol, 0.01 eq.), and 0.5 ml of dry NMP. The mixture was stirred for 5 min then sealed with a screw cap with a septum and transferred into the photoreactor (30 W 450 nm LED). After 15 h of irradiation under stirring, the mixture was diluted with 7 ml of water and extracted with Et_2O (3 x 4 ml). The combined organic fractions were washed with brine and dried over anhydrous Na_2SO_4, and the solvent was removed under reduced pressure. The residue was purified using column chromatography.

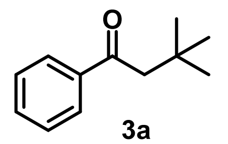

3a

3,3-Dimethyl-1-phenylbutan-1-one (3a). The **3a** was obtained from **1a** and **2a** as a colorless oil (photocatalyst/yield: Phz1/60%, Phz2/96%; eluent Hex/DCM 2:1, R_f = 0.2).

^{1}**H NMR** ($CDCl_3$): δ 7.98 – 7.88 (m, 2H), 7.57 – 7.50 (m, 1H), 7.49 – 7.39 (m, 2H), 2.86 (s, 2H), 1.07 (s, 9H).

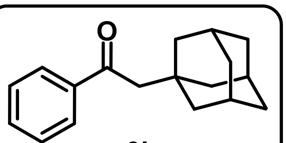

3b

2-(Adamantan-1-yl)-1-phenylethan-1-one (3b). The **3b** was obtained from **1a** and **2b** as a colorless oil (photocatalyst/yield: Phz1/53%, Phz2/73%; eluent Hex/DCM 2:1, R_f = 0.2).

^{1}**H NMR** ($CDCl_3$): δ 7.97 – 7.92 (m, 2H), 7.57 – 7.51 (m, 1H), 7.47 – 7.41 (m, 2H), 2.72 (s, 2H), 1.97 – 1.91 (m, 3H), 1.71 – 1.58 (m, 12H).

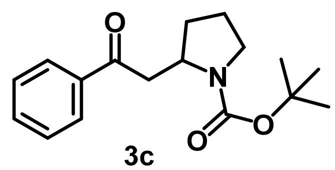

3c

Tert-butyl 2-(2-oxo-2-phenylethyl)pyrrolidine-1-carboxylate (3c). The **3c** was obtained from **1a** and **2c** as a yellowish oil (photocatalyst/yield: Phz1/56%, Phz2/61%; eluent Hex/EtOAc 15:1, R_f = 0.3).

^{1}**H NMR** ($CDCl_3$): δ 8.07 – 7.92 (m, 2H), 7.61 – 7.50 (m, 1H), 7.49 – 7.39 (m, 2H), 4.36 – 4.28 (m, 1H), 3.82 – 3.45 (m, 1H), 3.44 – 3.24 (m, 2H), 2.92 – 2.74 (m, 1H), 2.13 – 1.97 (m, 1H), 1.92 – 1.68 (m, 3H), 1.50 – 1.40 (m, 9H).

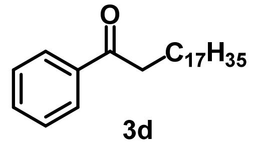

3d

1-Phenylnonadecan-1-one (3d). The **3d** was obtained from **1a** and **2d** as a white solid (photocatalyst/yield: Phz1/63%, Phz2/95%; eluent Hex/DCM 3:1, R_f = 0.3).

^{1}H NMR ($CDCl_3$):δ 7.99 – 7.93 (m, 2H), 7.58 – 7.52 (m, 1H), 7.48 – 7.42 (m, 2H), 2.99 – 2.92 (m, 2H), 1.78 – 1.68 (m, 2H), 1.42 – 1.19 (m, 30H), 0.91 – 0.85 (m, 3H).

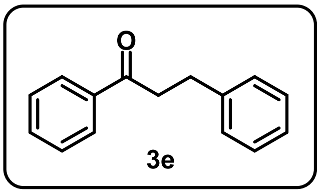

*1,3-Diphenylpropan-1-one (**3e**).* The **3e** was obtained from **1a** and **2e** as a colorless oil (photocatalyst/yield: Phz1/30%, Phz2/47%; eluent Hex/DCM 2:1, R_f = 0.2).

^{1}H NMR ($CDCl_3$): δ 8.00 – 7.95 (m, 2H), 7.60 – 7.54 (m, 1H), 7.50 – 7.44 (m, 2H), 7.35 – 7.19 (m, 5H), 3.35 – 3.29 (m, 2H), 3.12 – 3.06 (m, 2H).

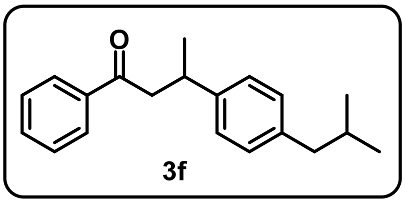

*3-(4-Isobutylphenyl)-1-phenylbutan-1-one (**3f**).* The **3f** was obtained from **1a** and **2f** as a colorless oil (photocatalyst/yield: Phz1/38%, Phz2/35%; eluent Hex/DCM 2:1, R_f = 0.2).

^{1}H NMR ($CDCl_3$): δ 7.95 – 7.91 (m, 2H), 7.57 – 7.52 (m, 1H), 7.46 – 7.42 (m, 2H), 7.20 – 7.16 (m, 2H), 7.10 – 7.06 (m, 2H), 3.53 – 3.43 (m, 1H), 3.29 (dd, J = 16.3, 5.5 Hz, 1H), 3.17 (dd, J = 16.3, 8.5 Hz, 1H), 2.44 (d, J = 7.2 Hz, 2H), 1.89 – 1.78 (m, 1H), 1.33 (d, J = 6.9 Hz, 3H), 0.89 (d, J = 6.6 Hz, 6H).

^{13}C NMR ($CDCl_3$): δ 199.28, 143.75, 139.56, 137.28, 132.88, 132.86, 129.20, 128.50, 128.07, 126.49, 47.22, 45.02, 35.28, 30.18, 22.39, 21.83.

HRMS (APCI-TOF): m/z = 281.1901 ([M+H]+, 281.1900 calculated for $C_{20}H_{25}O^{+}$).

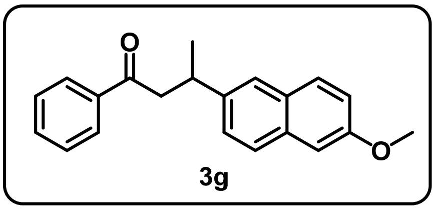

*3-(6-Methoxynaphthalen-2-yl)-1-phenylbutan-1-one (**3g**).* The **3g** was obtained from **1a** and **2g** as a colorless oil (photocatalyst/yield: Phz1/40%, Phz2/31%; eluent Hex/DCM 2:1, R_f = 0.15).

^{1}H NMR ($CDCl_3$): δ 7.95 (m, 2H), 7.72 – 7.67 (m, 2H), 7.63 (d, J = 1.4 Hz, 1H), 7.57 – 7.52 (m, 1H), 7.47 – 7.42 (m, 2H), 7.40 (dd, J = 8.5, 1.8 Hz, 1H), 7.15 – 7.10 (m, 2H), 3.91 (d, J = 1.8 Hz, 3H), 3.71 – 3.61 (m, 1H), 3.39 (dd, J = 16.4, 5.7 Hz, 1H), 3.26 (dd, J = 16.4, 8.2 Hz, 1H), 1.42 (d, J = 6.9 Hz, 3H).

^{13}C NMR ($CDCl_3$): δ 199.09, 157.31, 141.72, 137.24, 133.27, 132.93, 129.10, 129.06, 128.55, 128.07, 127.01, 126.18, 124.80, 118.74, 105.64, 55.32, 55.25, 47.08, 35.52, 21.91.

HRMS (APPI-TOF): m/z = 304.1458 ($M^{•+}$, 304.1458 calculated for $C_{21}H_{20}O_2^{•+}$).

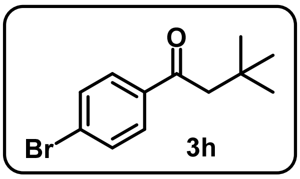

*1-(4-Bromophenyl)-3,3-dimethylbutan-1-one (**3h**).* The **3h** was obtained from **1b** and **2a** as a colorless oil (photocatalyst/yield: Phz1/85%, Phz2/91%; eluent Hex/DCM 2:1, R_f = 0.2).

^{1}H NMR ($CDCl_3$): δ 7.82 – 7.77 (m, 2H), 7.61 – 7.56 (m, 2H), 2.81 (s, 2H), 1.05 (s, 9H).

2. ESR measurements of photoexcited triplet states

The **ESR spectra** were recorded using a Bruker EMX plus X-band spectrometer at (100±0.5) K. The temperature of the samples was controlled using a flow of nitrogen gas. To obtain the spectra of photoinduced species, the samples were irradiated with a solid-state-diode laser at 405 nm. The irradiating beam entered the tube through the optical window and propagated from the top of ESR tube down inside the ESR cavity. The intensity of light was measured independently using ferrioxalate actinometry to be $5 \cdot 10^{-9}$ Einstein/s per sample. The background light-off spectra obtained in the same conditions were subtracted from the spectra recorded under light irradiation. The former was attributed to the background ESR signal of the cavity and the minor admixture of radical cations of the corresponding dihydrophenazines.

For **sample preparation** for the ESR measurements, the solutions of dihydrophenazines in toluene were placed in the ad hoc quartz tube equipped with an optical quartz window on the top of the tube (Fig. S4). The concentration of the solution was taken to provide a ca. 0.5 optical density of the 10 mm-long sample.

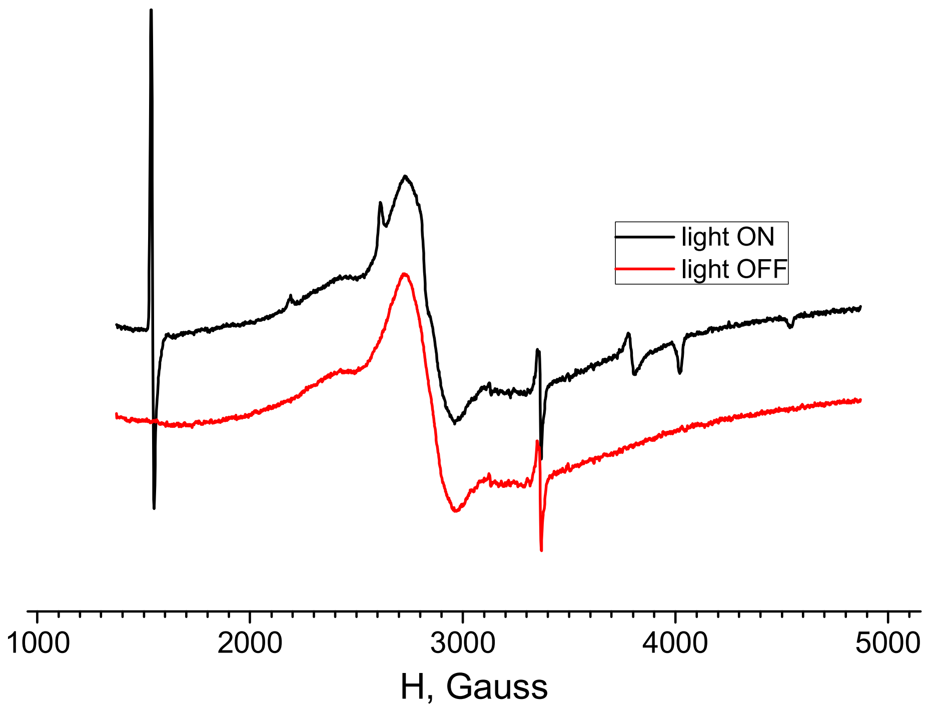

Figure S1. ESR spectrum of **Phz1** in toluene (100 K) recorded during continuous irradiation of the sample (black line) and in the absence of irradiation (red line). The broad line in the background spectrum was attributed to the empty resonator, whereas the sharper line at 3350G was attributed to the admixture of the **Phz1** radical cation in the sample.

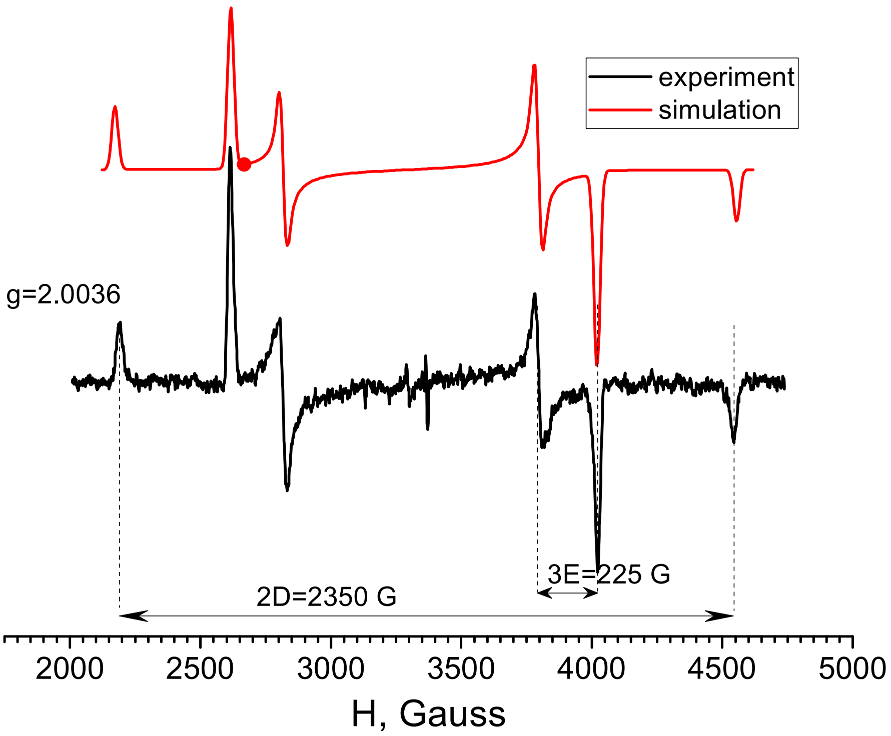

Figure S2. Experimental (black line) and simulated (red line) spectrum of the photoexcited triplet state of **Phz1** in toluene at 100K.

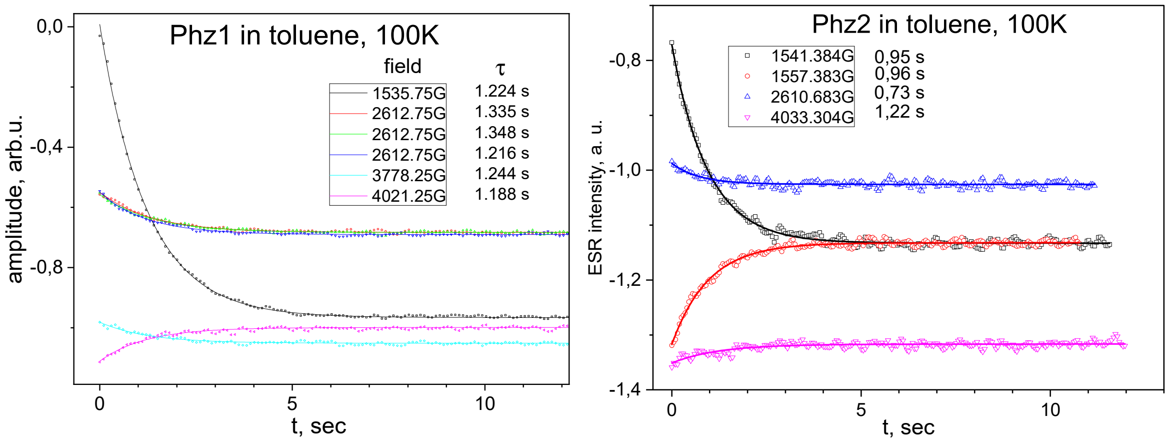

Figure S3. The time dependence of the ESR signal for the triplet forms of **Phz1** (a) and **Phz2** (b) in toluene at 100 K recorded in a constant magnetic field after turning off the irradiating light (points) and their best exponential fits (lines). The values of the magnetic field corresponding to each curve are shown in the legend.

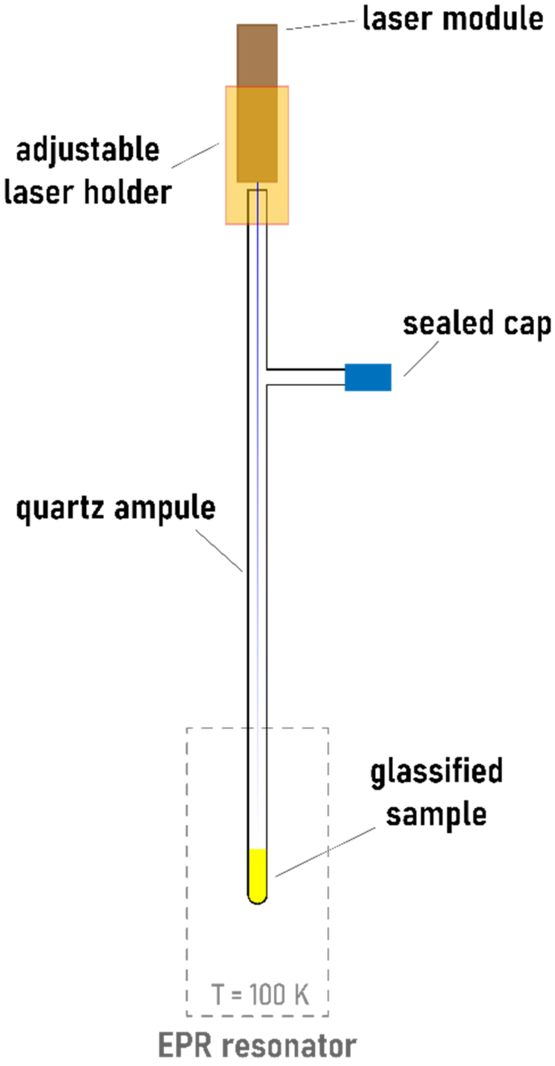

Figure S4. The ad hoc quartz EPR cell used in the experiments.

3. Photophysical experiments

The fluorescence lifetimes were measured with a Horiba Fluoromax instrument using the "lifetime decay" procedure. The raw data were fitted with an exponential decay function consisting of two exponents: one for the lamp decay with fixed parameters and one for the luminescence decay.

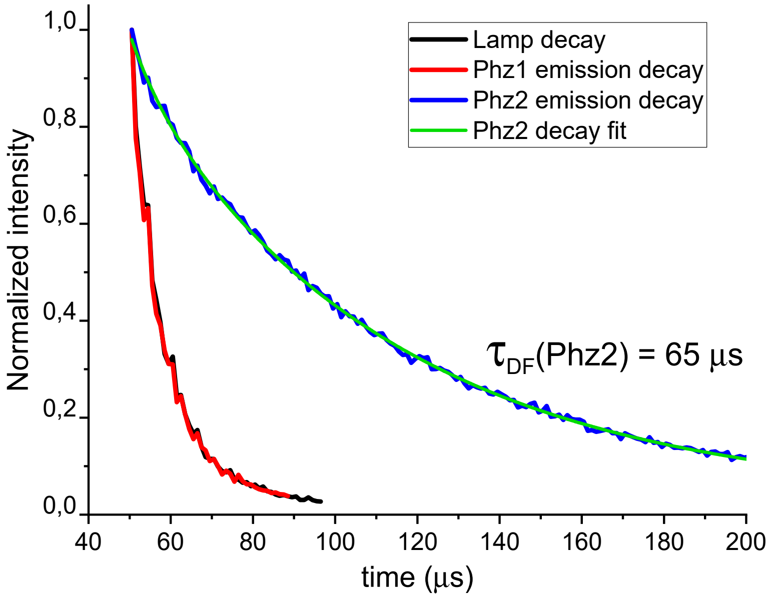

Figure S5. Time dependence of fluorescence intensity for deaerated solutions of **Phz1** and **Phz2** in toluene.

The phosphorescence lifetimes were measured as follows. An ampule with 100 l of Phz1 or Phz2 solution was placed in a quartz dewar filled with liquid nitrogen. The resulting set was placed in an Avaspec cuvette holder (Fig. S6). The sample was irradiated with a 380 nm LED for 2.0 sec; after a delay, the luminescence of the sample was integrated for 0.5 sec. This was repeated for the integration delay values from 0 to 4 sec with a step of 0.2 sec (Fig. S7). The resulting data (integral intensity vs. integration delay) were fitted with an exponential decay function to find the phosphorescence lifetimes (Figs. S8 and S9).

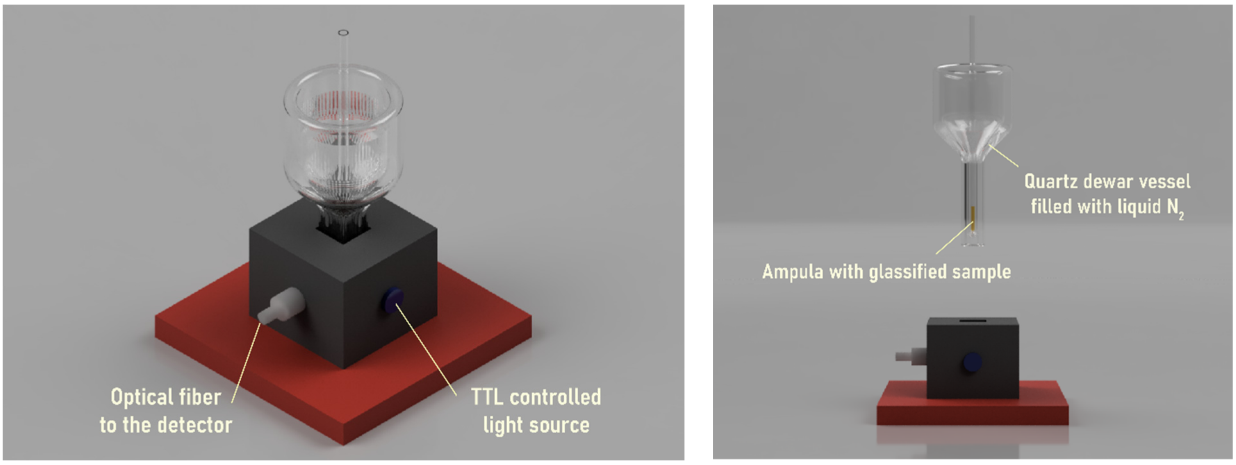

Figure S6. Experimental setup for the phosphorescence lifetime measurement.

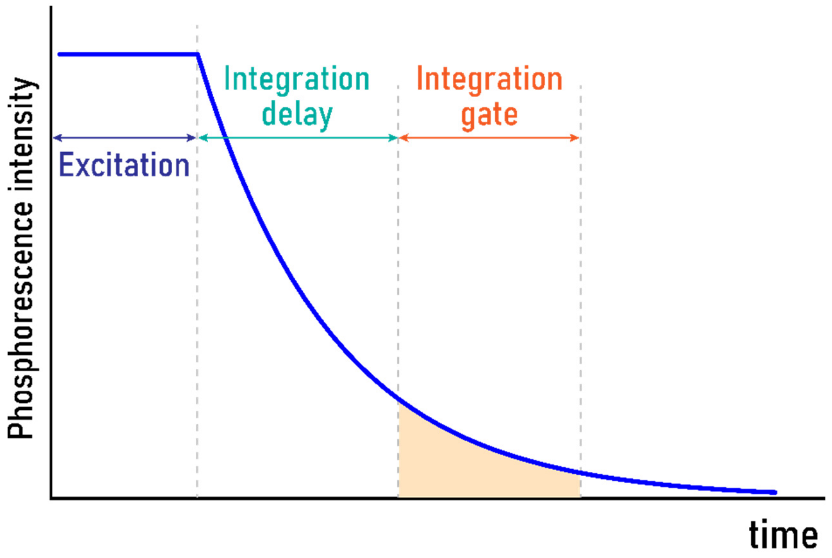

Experimental parameters

Excitation time	2 sec
Integration delay	0 to 4 sec
Delay step	0.2 sec
Integration gate	0.5 sec

Figure S7. Experimental scheme for the phosphorescence lifetime measurement.

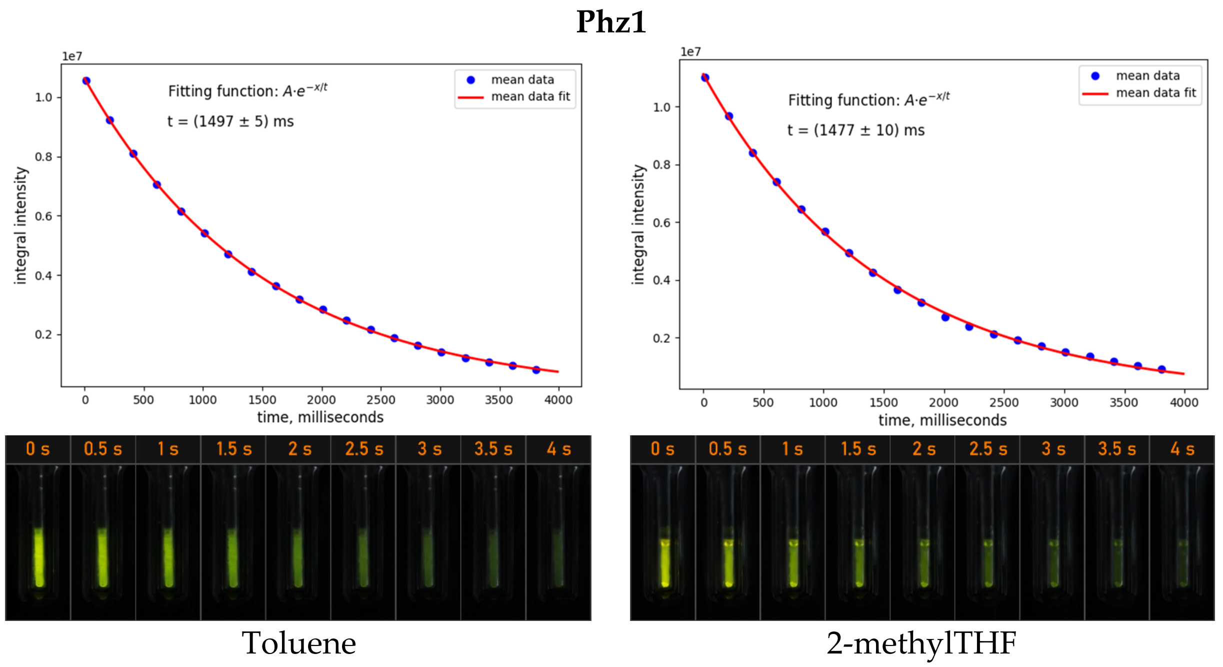

Figure S8. Time dependence of the integral phosphorescence intensity for glassified solutions of **Phz1** (top); photos of samples in a quartz dewar at regular intervals after turning off the excitation source (bottom).

Phz2

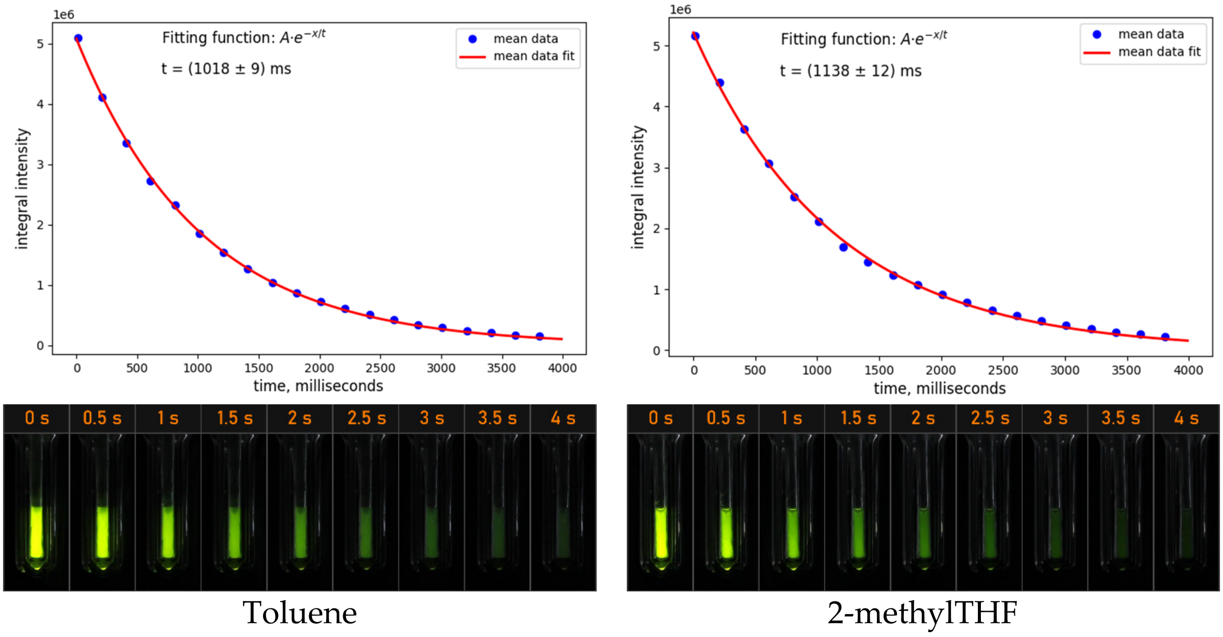

Figure S9. Time dependence of the integral phosphorescence intensity for glassified solutions of **Phz2** (top); photos of samples in a quartz dewar at regular intervals after turning off the excitation source (bottom).

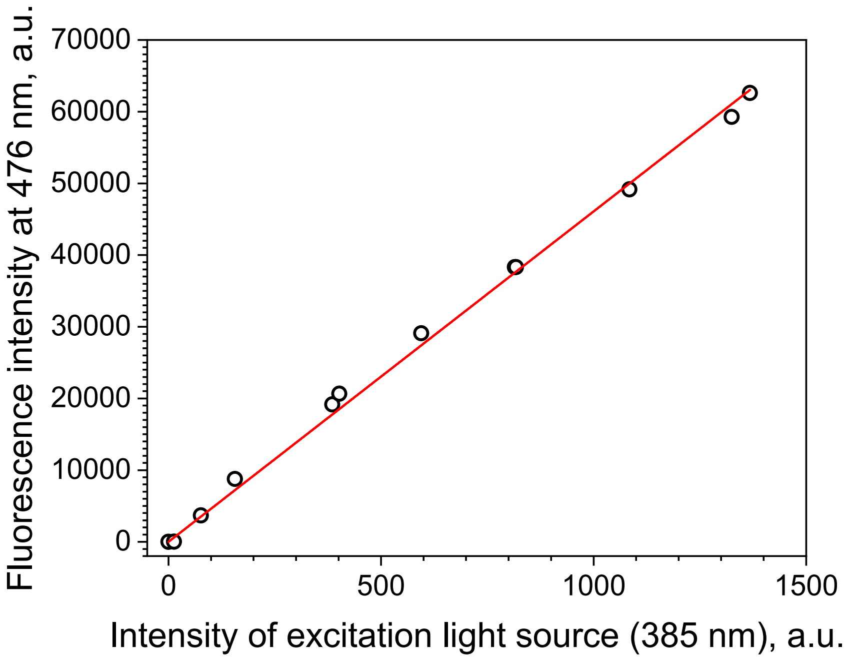

Figure S10. Fluorescence intensity vs. the power of the excitation light source for the toluene solution of **Phz1**

Table S1. Photophysical parameters for **Phz1** and **Phz2** determined based on spectral data.

	**Phz1 (Toluene)**		**Phz2 (Toluene)**	
	**eV**	**nm**	**eV**	**nm**
$_{max}$(abs)	3.3	375	3.37	370
$_{ons}$(abs)	2.98	416	2.84	436
$_{max}$(fluorescence)	2.6	476	2.37	523
$_{ons}$(fluorescence)	2.95	420	2.85	435
$_{max}$(phosphorescence)	2.2	566	2.21	561
$_{ons}$(phosphorescence)	2.51	496	2.52	492
E_{00}(S1) intersection	2.95	421	2.79	444
E_{00}(S1) from onsets	2.96	419	2.85	435
E_{00}(T1) from onsets	2.74	452	2.68	263
$E_{ST} = E_{00}$(S1) - E_{00}(T1)	0.22	-	0.11	-

4. Experimental setup for photochemical experiments

All photoredox-catalyzed reactions were carried out using the ad hoc photoreactor constructed with acrylic panels with a mirror side turned inward. As a light source, two LED panels were used (2x15 W, 450 nm blue light). The top panel contained a vial holder for four positions. The reaction mixtures were stirred using an IKA RCT Basic device placed underneath.

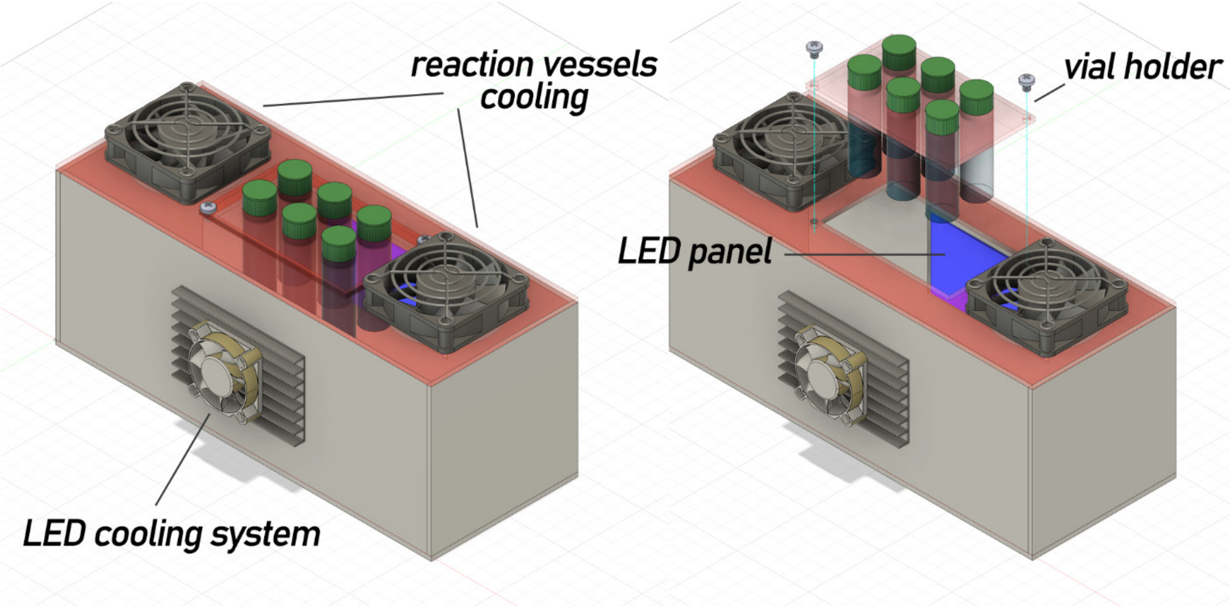

Figure S11. The 3D model of the photoreactor.

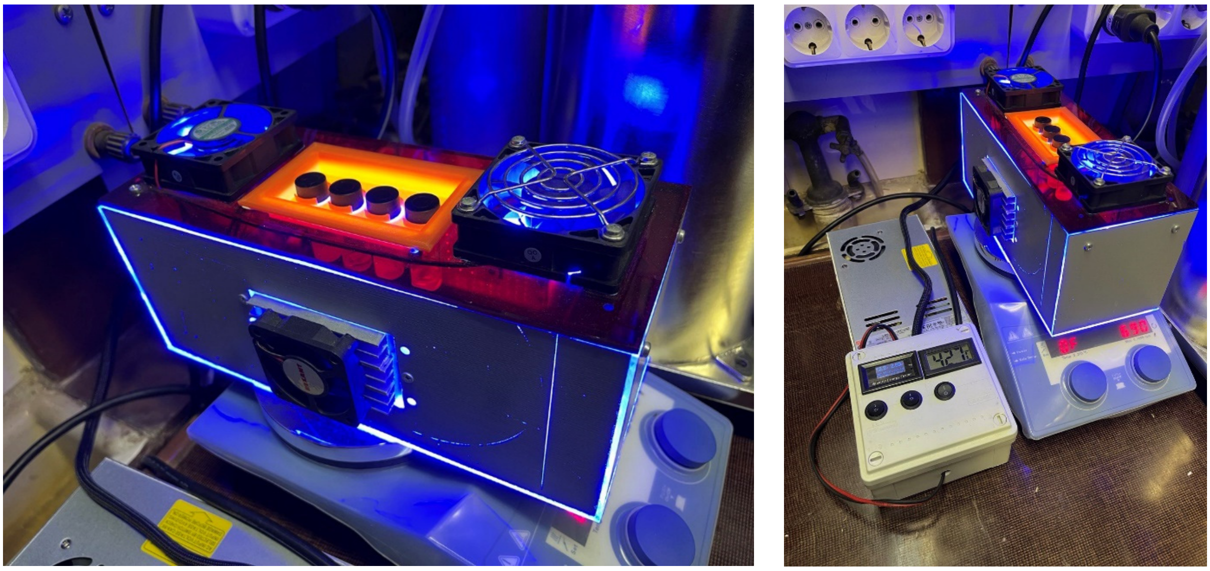

Figure S12. The photoreactor at work (left); the complete experimental setup containing a 12V DC power supply, the control block, the photoreactor, and the IKA RCT basic magnetic stirrer (right).

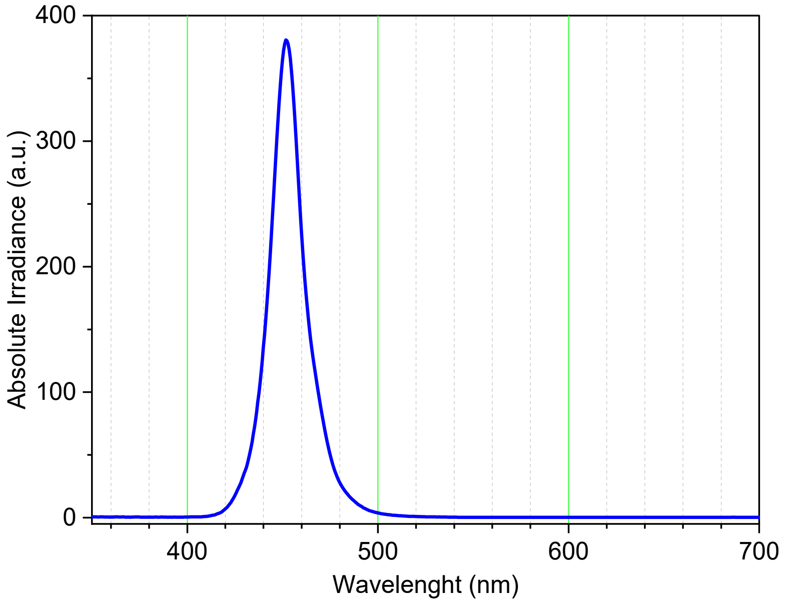

Figure S13. The spectrum of the light source used in the photoreactor.

5. Electrochemical measurements

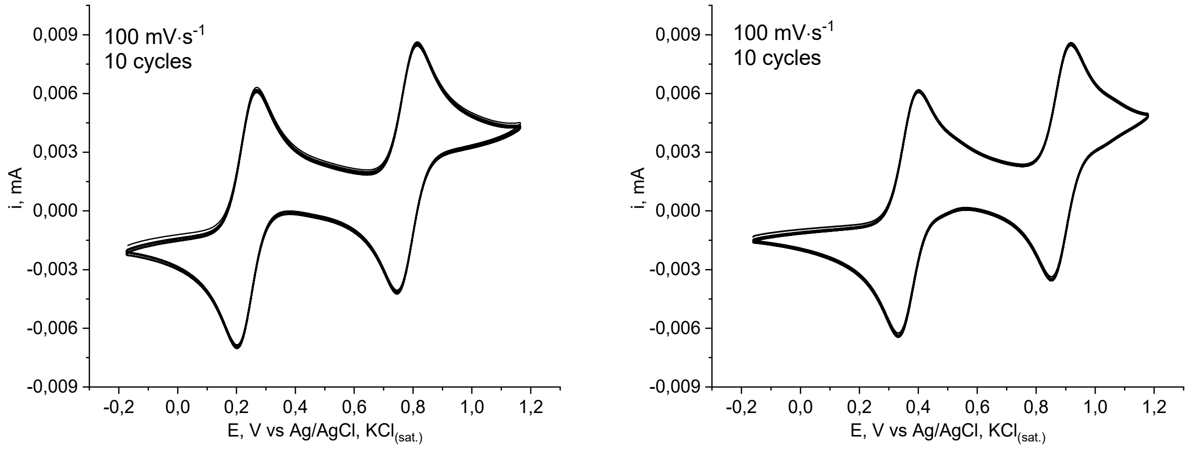

Figure S14. Cyclic voltammograms for **Phz1** (left) and **Phz2** (right) (Pt, 100 mV/s, THF, 0.2 M Bu_4NBF_4, 10 cycles).

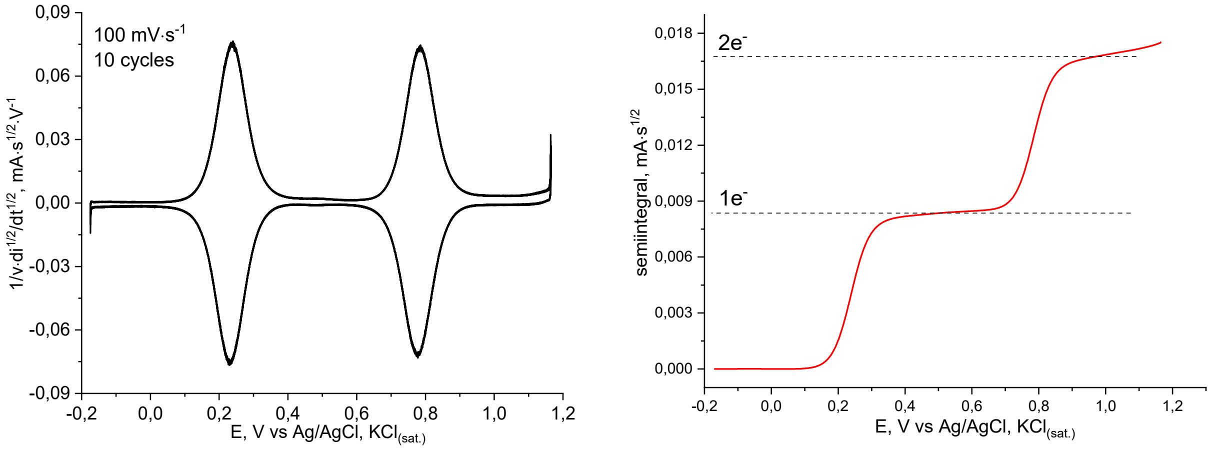

Figure S15. Semi-differential (left) and semi-integral (right) voltammograms for **Phz1** (Pt, 100 mV/s, THF, 0.2 M Bu_4NBF_4).

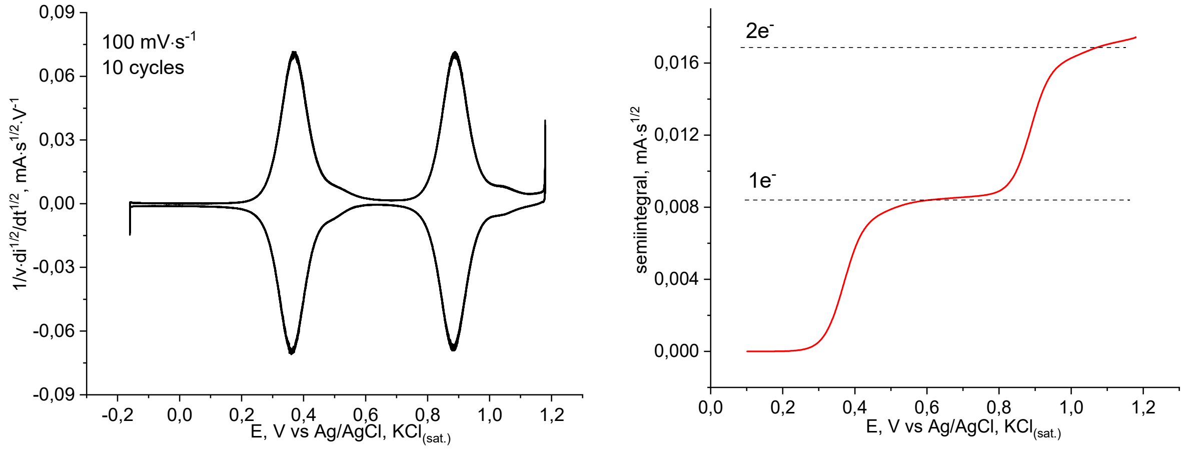

Figure S16. Semi-differential (left) and semi-integral (right) voltammograms for **Phz2** (Pt, 100 mV/s, THF, 0.2 M Bu_4NBF_4).

Table S2. Electrochemical characteristics of **Phz1** and **Phz2**.

	**Phz1**	**Phz2**
$E_{1/2}$(1st wave), V vs Ag/AgCl	0.24	0.37
$E_{1/2}$(2nd wave), V vs Ag/AgCl	0.78	0.89
EC gap, V	0.54	0.52

6. Computational data

To calculate the E_{ST} values for Phz1 and Phz2, an exchange integral between the HOMO and LUMO was calculated for both systems ($E_{ST} = 2J_{HOMO\text{-}LUMO}$).

$$J = \iint \varphi_{HOMO}(r_1)\varphi_{LUMO}(r_2)\frac{e^2}{|r_1 - r_2|}\varphi^*{}_{HOMO}(r_2)\varphi^*{}_{LUMO}(r_1)dr_1 dr_2$$

The obtained data are presented in the table below.

Table S3. E_{ST} values for **Phz1** and **Phz2** calculated using different functionals. The values are given in eV.

**Phz1**	**Phz2**
0.26 (PBEh-3c)	0.01 (PBEh-3c)
0.20 (PBE0)	0.009 (PBE0)
0.19 (B3LYP)	0.009 (B3LYP)

Cartesian coordinates for the optimized geometry of **Phz1**.

C	-1.17976219344198	3.53986720144433	0.00393591749608
C	-2.37399255092103	2.83427488296086	-0.01032488454323
C	-2.38731553949579	1.44303942771075	-0.01292999243181
C	-1.20919051021188	0.70830331345875	-0.00431242118001
C	0.01175773226850	1.40518398693036	0.00479059534014
C	0.00350839218727	2.79551985113765	0.01073968391689
N	1.20802695593845	0.68497080617728	0.00943441458433
C	1.21128825729013	-0.71170353729229	0.00770954180292
C	-0.00966053094557	-1.40857752158040	-0.00144428745627
N	-1.20593086266905	-0.68836727436359	-0.00312619301222
C	-2.43667560934345	-1.38607142504533	0.01824564025174
C	-3.06242135091363	-1.75413917968202	-1.16931985302852
C	-4.25888220949791	-2.45084340701993	-1.14015017836953
C	-4.87448188690758	-2.80462478494647	0.06500086782945
C	-4.23527750510942	-2.42422035875769	1.24380482521952
C	-3.03383264250296	-1.72583385463917	1.22384923581770
C	2.43877253009135	1.38267297077796	-0.01207786052570
C	3.03618767294838	1.72215111742948	-1.21769687527709
C	4.23761976185701	2.42047832615462	-1.23757572127918
C	4.87658775428380	2.80122273832117	-0.05872102714167
C	4.26075639885303	2.44773956600644	1.14636531122572
C	3.06427388380591	1.75100310038954	1.17547310712085
C	2.38941999091521	-1.44645099631258	0.01410045690202
C	2.37609601803810	-2.83767938961656	0.00950324474687
C	-0.00141734348713	-2.79890390314711	-0.00968479878295
C	1.18185755768334	-3.54325741856240	-0.00481735551613

C	1.17927884510606	-5.07154914115537	0.02235896102580
C	1.68907176449290	-5.54088684715513	1.39267852444792
C	-0.21899893387369	-5.65041830750635	-0.20067041208988
C	2.09742560410463	-5.61482648677422	-1.08047655627165
C	-1.17715652477296	5.06811994644388	-0.02519171801646
C	-2.09571231079195	5.61278567679593	1.07662460934192
C	0.22104952698641	5.64724122900016	0.19766691625103
C	-1.68642895343356	5.53580746831655	-1.39626578194180
C	-6.19108162312032	-3.57863525635698	0.04356124529479
C	-5.98026971832656	-4.90757727159540	-0.69604836778618
C	-6.70281669942564	-3.88910147995659	1.45052828351389
C	-7.25462574483668	-2.74572296370584	-0.68553874727414
C	6.19327717634182	3.57508130933050	-0.03719653290519
C	6.70500838333717	3.88564217390678	-1.44412127297527
C	5.98267017915089	4.90391519277437	0.70263795132228
C	7.25672183683821	2.74192548338156	0.69180784554996
H	-3.32900834706739	3.35779118350365	-0.02006453725380
H	-3.34287550185162	0.92314083897407	-0.02100267921434
H	0.95872643435245	3.31115454823992	0.02239966710716
H	-2.60071699254107	-1.49251241070969	-2.12042440143881
H	-4.71898993171763	-2.72784022491123	-2.08821899761008
H	-4.66563512129502	-2.67168278118609	2.21100131657929
H	-2.55075624139065	-1.44249599645572	2.15800718606638
H	2.55337237534353	1.43860068628478	-2.15193098957242
H	4.66817642358642	2.66766555871442	-2.20475868625862
H	4.72072472804251	2.72497543261011	2.09442879918481
H	2.60229458010492	1.48956204648335	2.12648914194600
H	3.34498463189666	-0.92656390973733	0.02227055464895
H	3.33111798484849	-3.36120765716169	0.01775667073339
H	-0.95665654373579	-3.31450439681907	-0.02127867995787
H	1.69949574948669	-6.63747513306851	1.45691710967829
H	2.70834180413173	-5.18846143958542	1.58955468586924
H	1.05147433303355	-5.16310599380263	2.20162464340182
H	-0.17414345124278	-6.74618115951051	-0.22525404989918
H	-0.91862805435697	-5.37750811921393	0.59871821106564
H	-0.65375418289146	-5.31988998340951	-1.15247320581276
H	2.09686398382794	-6.71289296404027	-1.07966175812396
H	1.76820320750669	-5.27897351203651	-2.07152534902778
H	3.13751569535159	-5.29292244374784	-0.95408088449086
H	-2.09521058905402	6.71085121751204	1.07435273263647
H	-3.13573562756337	5.29064335626754	0.95028521832288
H	-1.76680776813558	5.27822970566756	2.06821828116553
H	0.17621888125168	6.74303227324797	0.22094418923747
H	0.65545349173235	5.31778413151398	1.15000013162787
H	0.92095311641214	5.37336732791898	-0.60115407839958
H	-1.69677416375310	6.63231883304950	-1.46180080805407

H	-1.04857009359249	5.15700540896900	-2.20452654557679
H	-2.70565598414242	5.18321324760957	-1.59306650077662
H	-6.90803530495573	-5.49385493289435	-0.71132296810617
H	-5.67197897981241	-4.76060481259393	-1.73776752499031
H	-5.20834236247979	-5.51568870647969	-0.20826210703763
H	-7.64640347889063	-4.44550097862706	1.39177444581192
H	-5.99794705359523	-4.50660387785328	2.02102841453797
H	-6.90220111852324	-2.97928556055349	2.02995009437804
H	-8.20619898173707	-3.29083433797102	-0.73463904996921
H	-7.44078160210349	-1.79622091005345	-0.16868235160956
H	-6.96062203006907	-2.50701453272802	-1.71441439566966
H	7.64870084393188	4.44186052580891	-1.38535329646035
H	6.90419786244258	2.97588781784493	-2.02369747831615
H	6.00015974235376	4.50333043441170	-2.01445776923607
H	6.91044745969315	5.49019213964327	0.71758966019985
H	5.21056266925865	5.51214489096291	0.21529686710313
H	5.67484645797209	4.75677418934327	1.74446175560935
H	8.20836388375980	3.28690389890638	0.74110432368917
H	6.96254506601830	2.50309968421183	1.72061348387846
H	7.44284767166866	1.79248095377313	0.17483901718657

Cartesian coordinates for the optimized geometry of **Phz2**.

C	-1.19159712098740	3.53324947543412	0.03465721892801
C	-2.38289119338433	2.82257846857778	0.01725491110509
C	-2.39096300985195	1.43180545568924	0.01179172179562
C	-1.20993943844239	0.70194007397473	0.02040347054298
C	0.00732410705070	1.40313153285338	0.03351321219113
C	-0.00574917611085	2.79330315397653	0.04236087991140
N	1.20637506573402	0.68503272637652	0.03828341980746
C	1.21629596834641	-0.71312393785211	0.03680920314524
C	-0.00101553359996	-1.41435826220677	0.02371627834528
N	-1.19994337134667	-0.69631971979723	0.01747876811346
C	-2.42804238694657	-1.39502725533825	0.02759020971397
C	-3.04304344403684	-1.74902978325570	-1.16979428190426
C	-4.24515524467695	-2.43830345762897	-1.16041215516087
C	-4.84327439746829	-2.78002191820640	0.04962391545120
C	-4.23175012078246	-2.42675177752554	1.24755517836912
C	-3.02784982925600	-1.73760197498844	1.23443543669680
C	2.43384249096356	1.38438662180275	0.00985626985724
C	3.01995740780595	1.71720421304252	-1.20621478173778
C	4.22238808843731	2.40853368495591	-1.23847672431432
C	4.84683817575677	2.77136867918407	-0.05057874890086
C	4.26334652930950	2.43806626031047	1.16916902925989
C	3.06219878701627	1.74788476779934	1.19770514748350
C	2.39730243313165	-1.44295686298405	0.04651041102590

C	2.38927543860767	-2.83375326035759	0.04179255305825
C	0.01210278170153	-2.80453269790424	0.01576851384582
C	1.19800504450032	-3.54443668602279	0.02430155583587
C	1.20239184886503	-5.07252314938093	0.05175074221857
C	1.71139965771300	-5.53764372104994	1.42386915464300
C	-0.19238800655369	-5.65813853987265	-0.17442925909074
C	2.12620670043031	-5.61094234410762	-1.04879207387791
C	-1.19586647793804	5.06134583848698	0.00777969817709
C	-2.11950756589577	5.59935384340557	1.10867051502566
C	0.19898302146428	5.64679990057488	0.23393303069809
C	-1.70510159522220	5.52697521986592	-1.36407987628484
C	-6.17628915052526	-3.48182953382233	0.04496586449657
F	-6.25237551319625	-4.38224048183090	-0.92583564100522
F	-6.41473485374011	-4.10979643492820	1.18693545115470
F	-7.16902109802453	-2.62004404933920	-0.14475603734325
C	6.12939272289702	3.56125793811656	-0.06305176000587
F	6.74257575070548	3.50103492228897	-1.23590495962290
F	5.90435627802635	4.84384189827072	0.19779222824904
F	6.98196122141331	3.12212197401941	0.85328845587383
H	-3.33985554771625	3.34205380147759	0.00691228141777
H	-3.34627771273649	0.91148948726432	0.00054949118407
H	0.94555617605761	3.31628005890218	0.05706615065210
H	-2.56992586810528	-1.48139143489473	-2.11302223105755
H	-4.71018393412576	-2.70859545336135	-2.10707959864542
H	-4.68498080356212	-2.68543545811970	2.20241442431258
H	-2.54365303860970	-1.46130047350963	2.16952918548856
H	2.52750841167698	1.42975571073889	-2.13361529327421
H	4.66644667566978	2.65815698693657	-2.19997350879517
H	4.74184892115908	2.71186698906691	2.10831820142670
H	2.60166479002548	1.48382536148249	2.14814493334646
H	3.35254669118130	-0.92247994052572	0.05751369390314
H	3.34623009230878	-3.35324143853536	0.05265648367197
H	-0.93923040973243	-3.32747354328751	0.00070841703358
H	1.72680568095282	-6.63390797676779	1.48805146381736
H	2.72866964903938	-5.18084318144643	1.62305336306574
H	1.06996247320288	-5.16345135760965	2.23143611018935
H	-0.14156605404602	-6.75334497134038	-0.19960746405821
H	-0.89505946215320	-5.39141488215790	0.62445764847825
H	-0.62700677423093	-5.33070273563702	-1.12739607527097
H	2.13069596047756	-6.70875038386520	-1.04687029602657
H	1.79784517825035	-5.27809621184553	-2.04112213434170
H	3.16466827380611	-5.28478170003919	-0.92004236259095
H	-2.12386863697974	6.69715941586178	1.10724463255252
H	-3.15802243157418	5.27335263027275	0.97992561101963
H	-1.79103820707910	5.26605131517178	2.10081209153931
H	0.14829735249382	6.74199692113459	0.25940592510624

H	0.63370598931832	5.31916433486109	1.18677776965431
H	0.90169491614329	5.38031127137920	-0.56497898435068
H	-1.72040535036111	6.62326068976457	-1.42785772870754
H	-1.06379285928351	5.15306030509451	-2.17188065783729
H	-2.72243810335765	5.17033940292736	-1.56324964867434

7. HRMS spectra

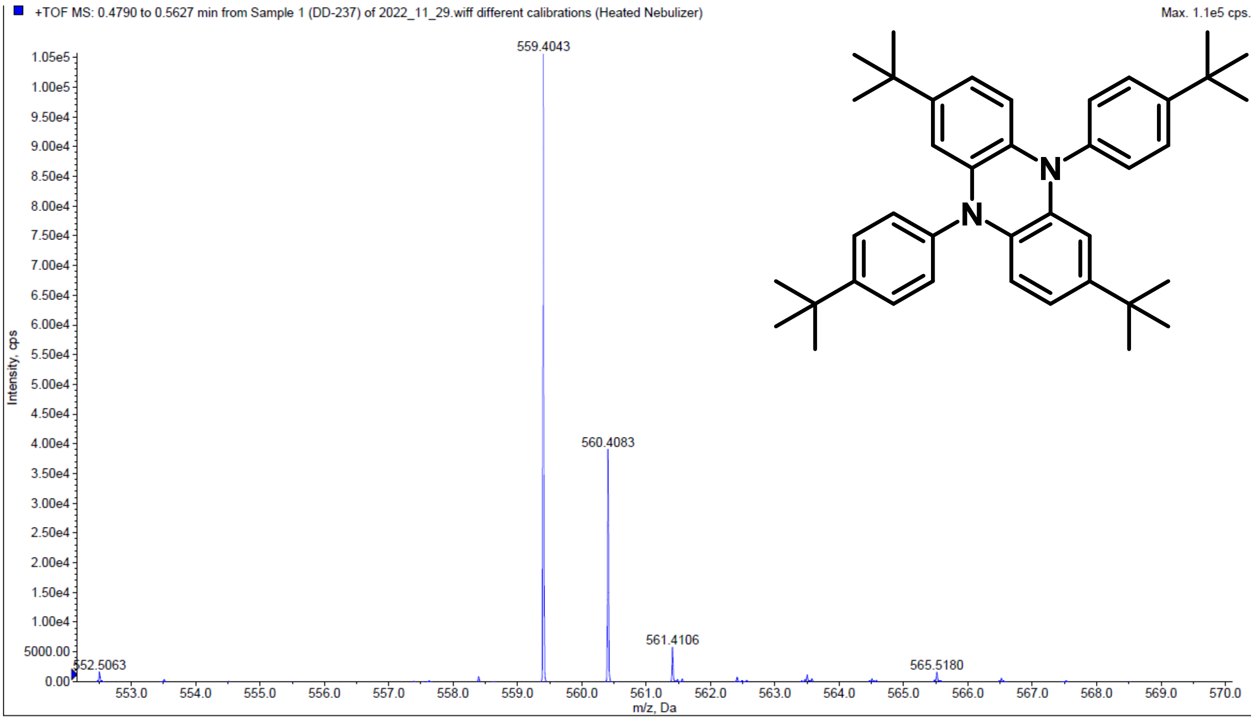

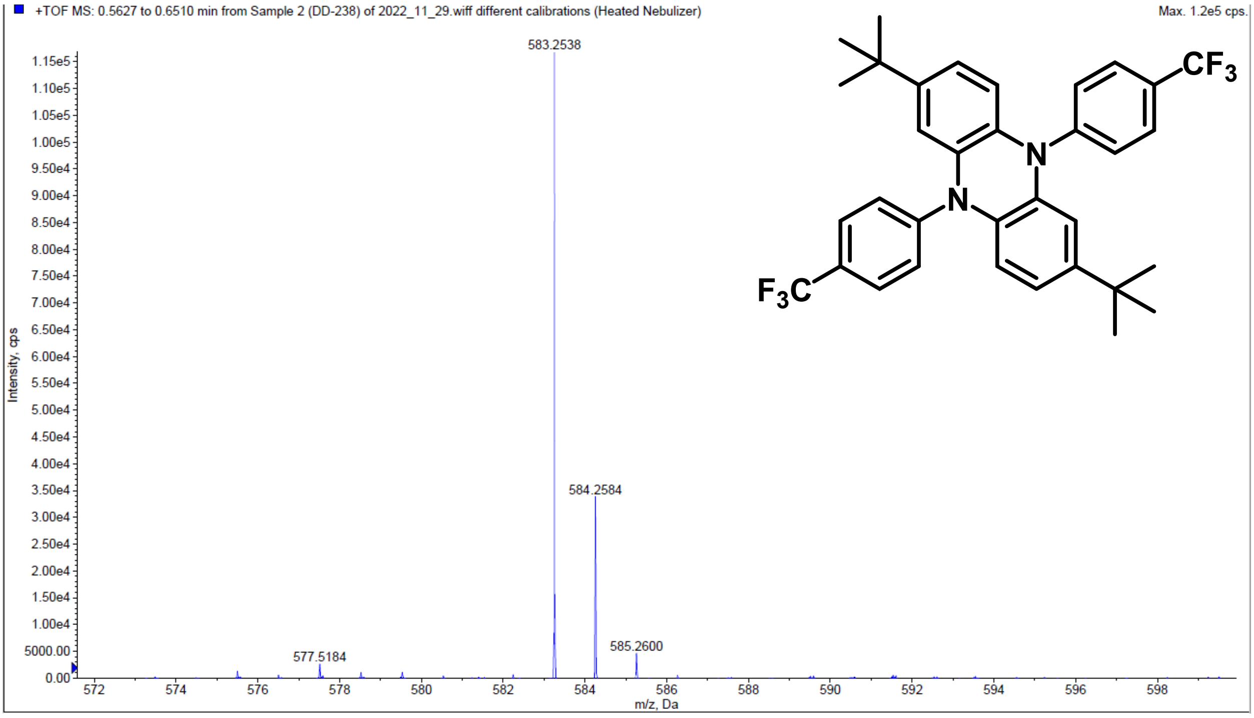

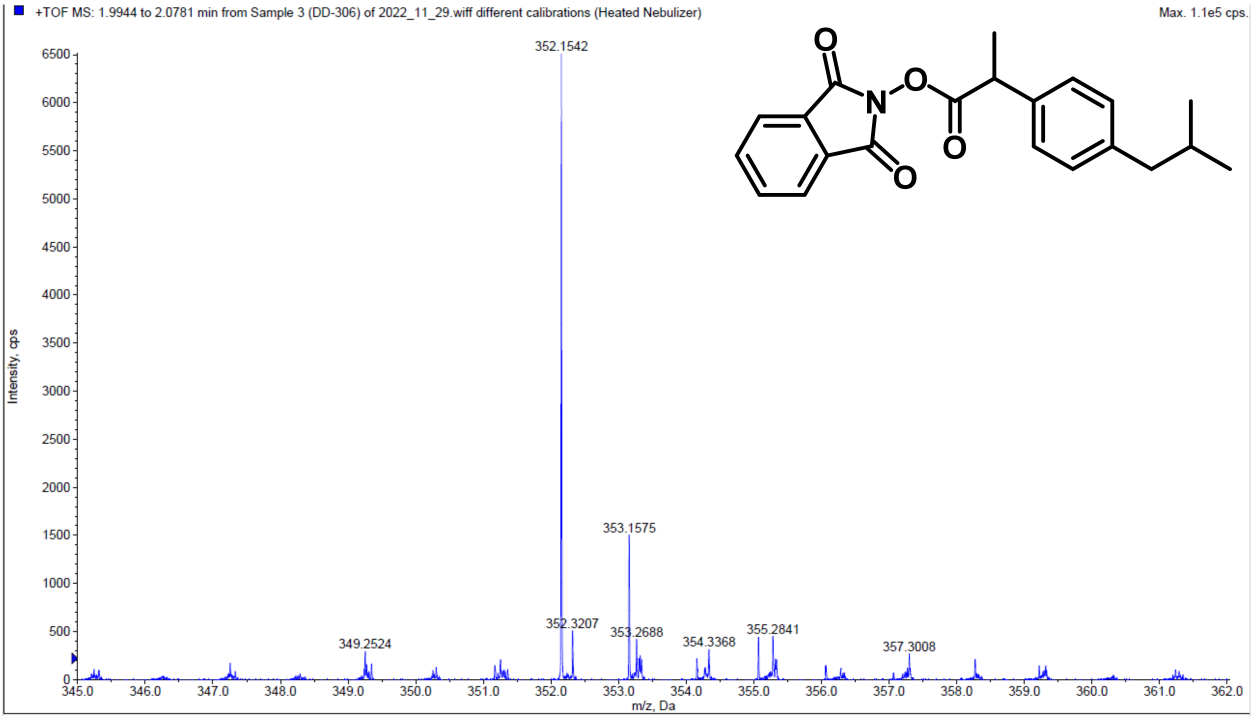

+TOF MS: 1.9944 to 2.0781 min from Sample 3 (DD-306) of 2022_11_29.wiff different calibrations (Heated Nebulizer)
Max. 1.1e5 cps.
352.1542
353.1575
352.3207
353.2688
354.3368
355.2841
349.2524
357.3008
Intensity, cps
m/z, Da

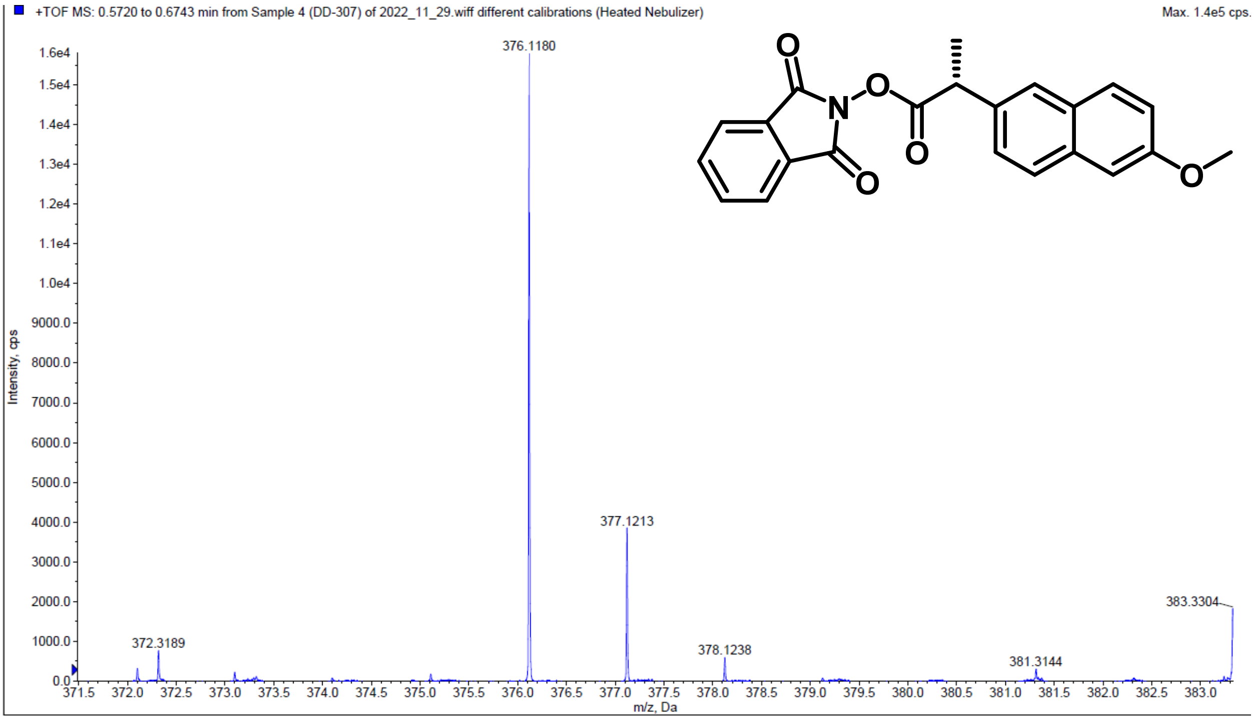

+TOF MS: 0.5720 to 0.6743 min from Sample 4 (DD-307) of 2022_11_29.wiff different calibrations (Heated Nebulizer)
Max. 1.4e5 cps.
376.1180
377.1213
383.3304
372.3189
378.1238
381.3144
Intensity, cps
m/z, Da

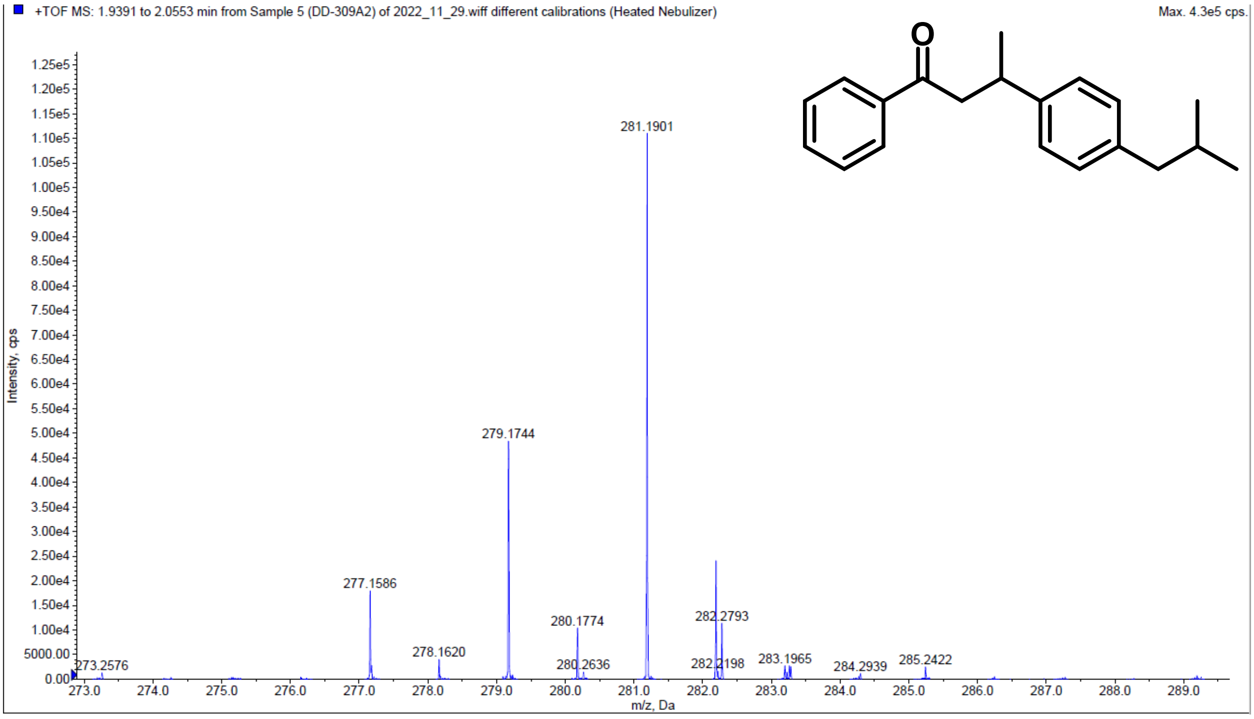
+TOF MS: 1.9391 to 2.0553 min from Sample 5 (DD-309A2) of 2022_11_29.wiff different calibrations (Heated Nebulizer)
Max. 4.3e5 cps.
Intensity, cps
m/z, Da
281.1901
279.1744
277.1586
280.1774
282.2793
278.1620
273.2576
280.2636
282.2198
283.1965
284.2939
285.2422
O

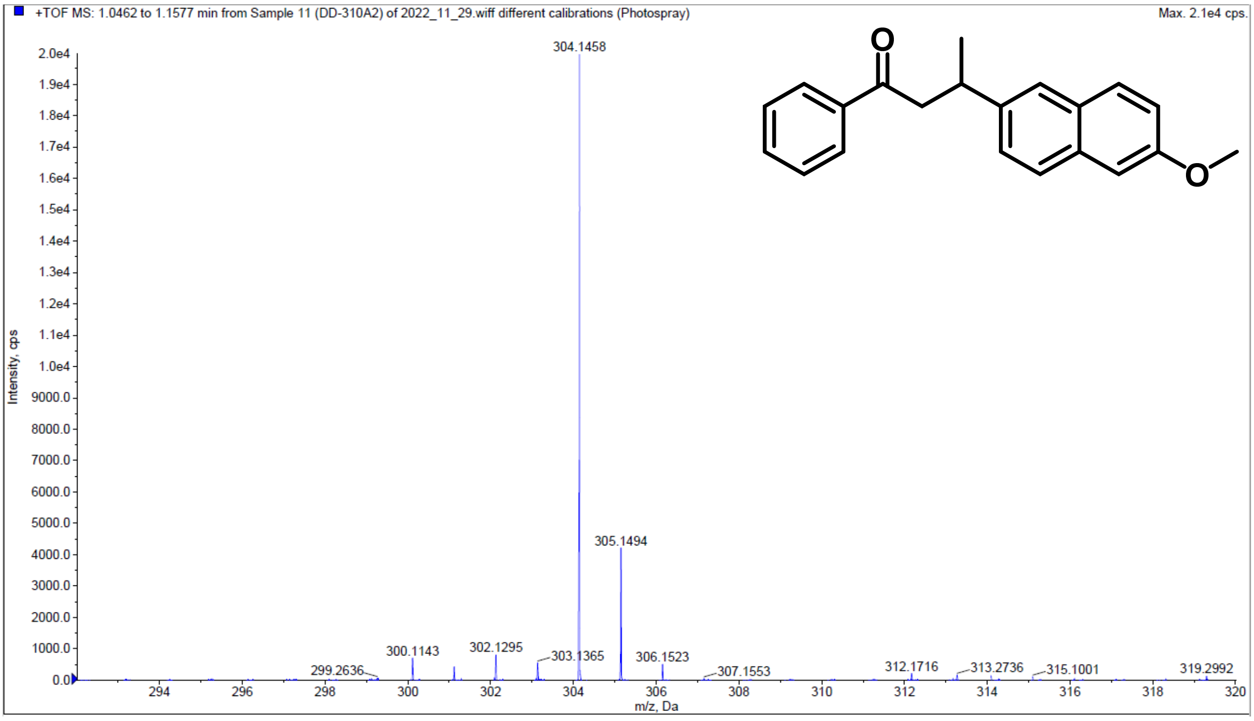
+TOF MS: 1.0462 to 1.1577 min from Sample 11 (DD-310A2) of 2022_11_29.wiff different calibrations (Photospray)
Max. 2.1e4 cps.
Intensity, cps
m/z, Da
304.1458
305.1494
300.1143
302.1295
299.2636
303.1365
306.1523
307.1553
312.1716
313.2736
315.1001
319.2992
O
O

8. NMR spectra

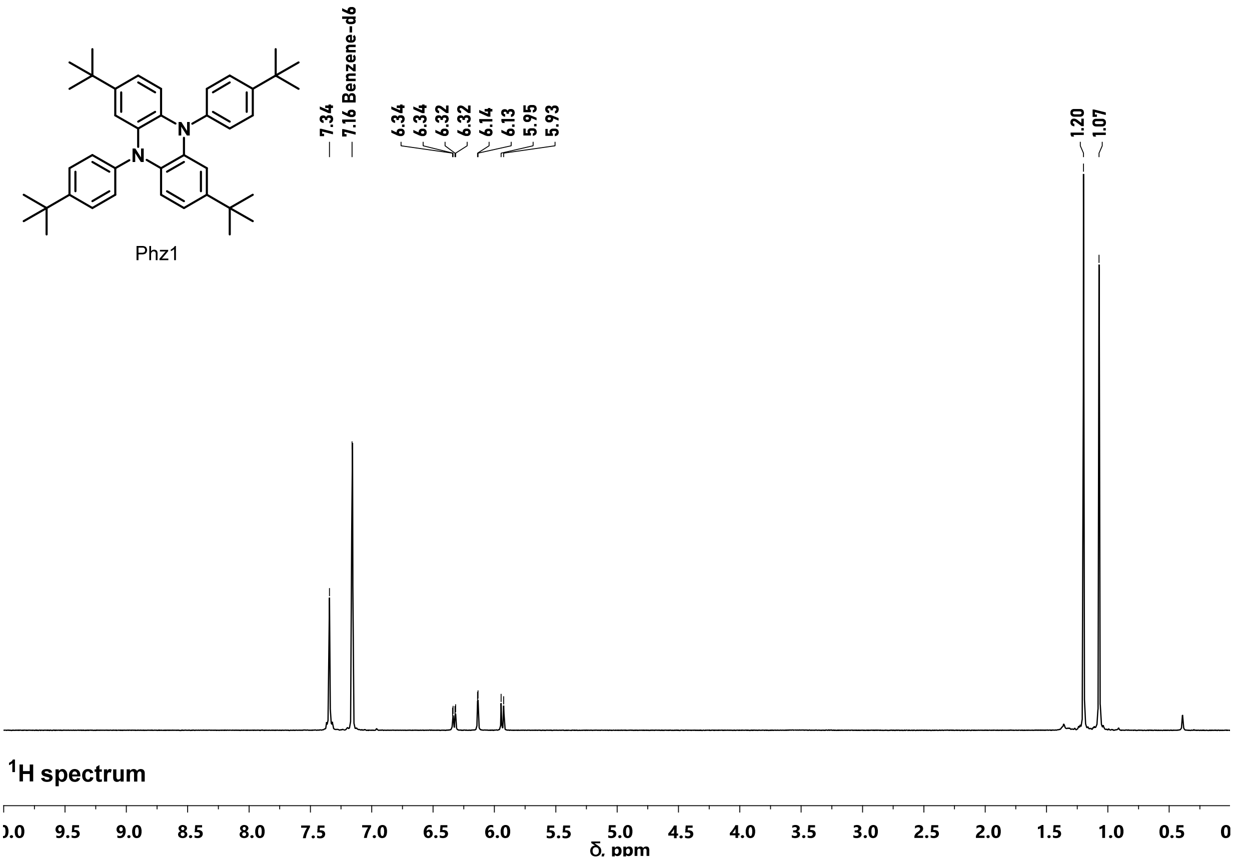

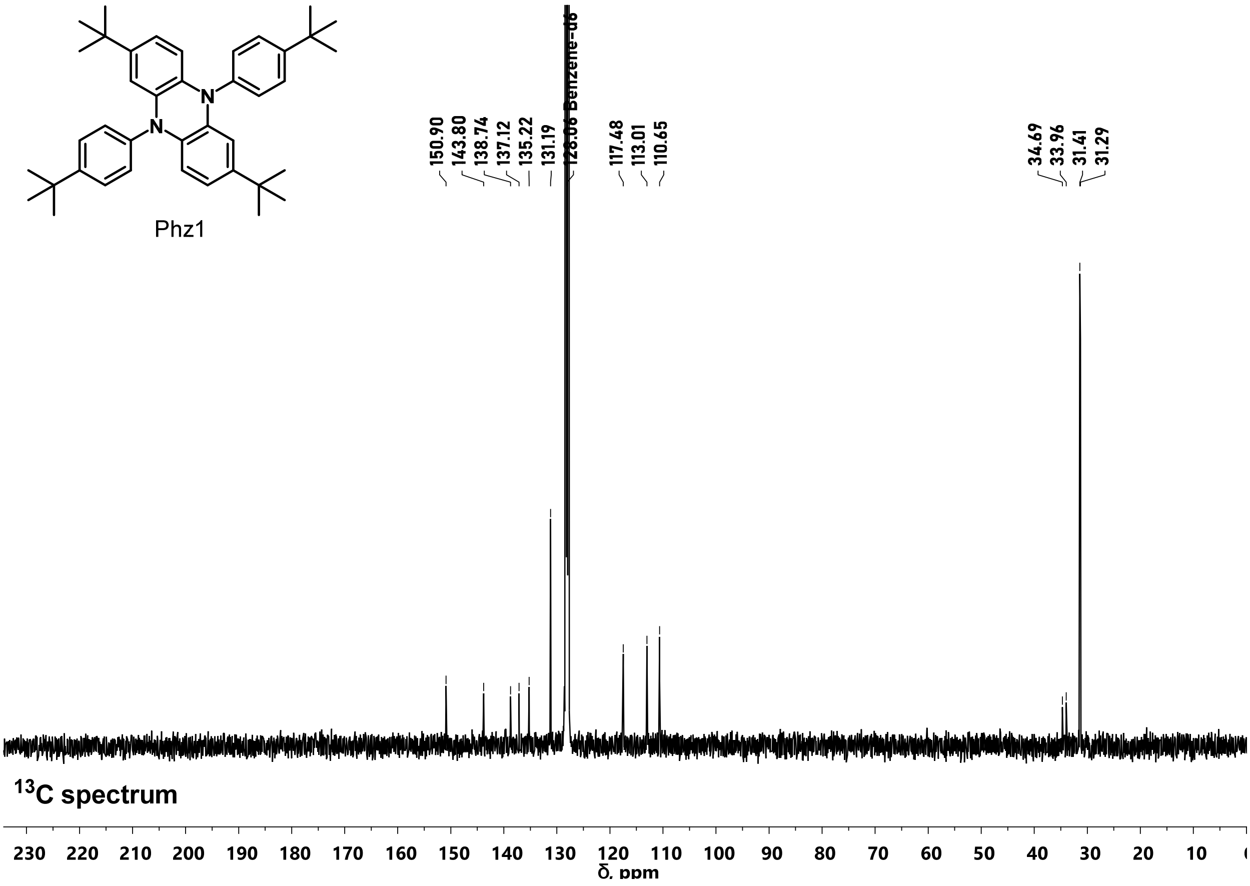

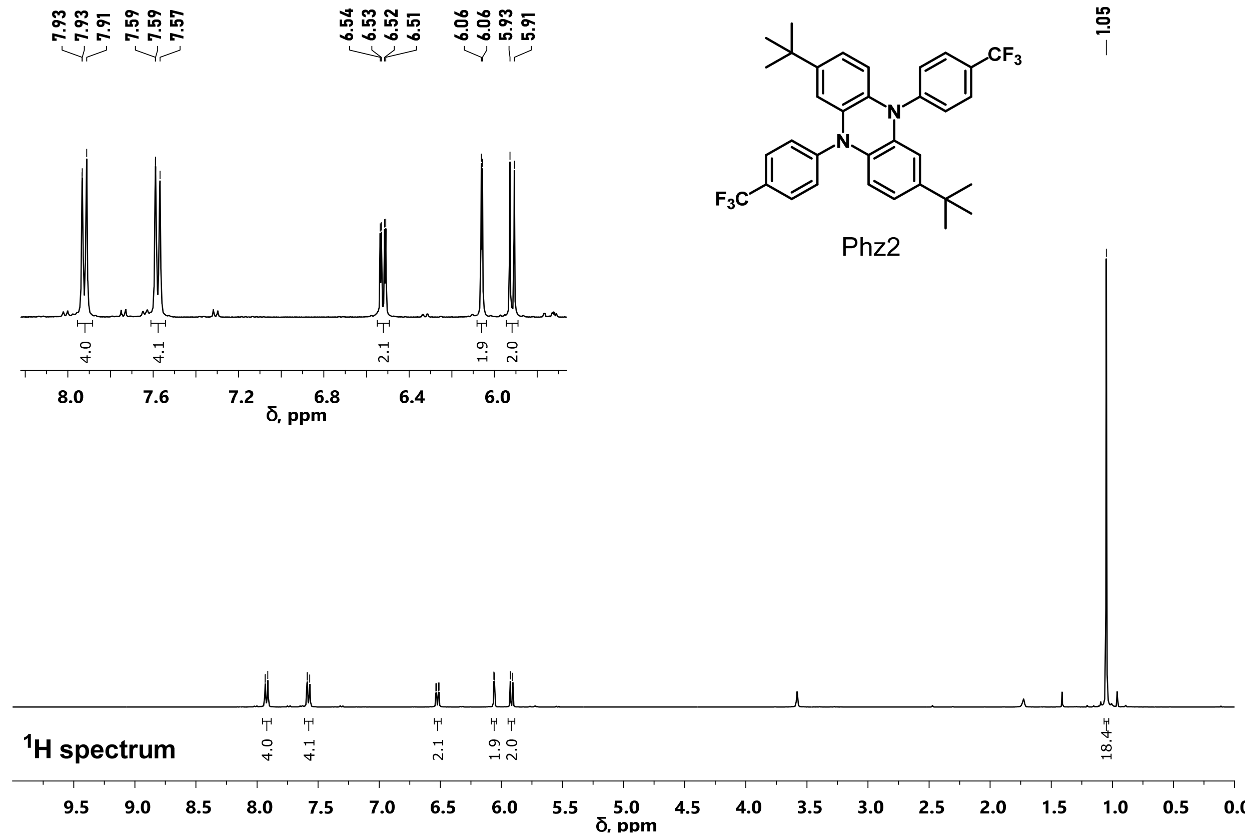
7.93
7.93
7.91
7.59
7.59
7.57
6.54
6.53
6.52
6.51
6.06
6.06
5.93
5.91
1.05
CF3
N
N
F3C
Phz2
4.0
4.1
2.1
1.9
2.0
18.4
δ, ppm
1H spectrum

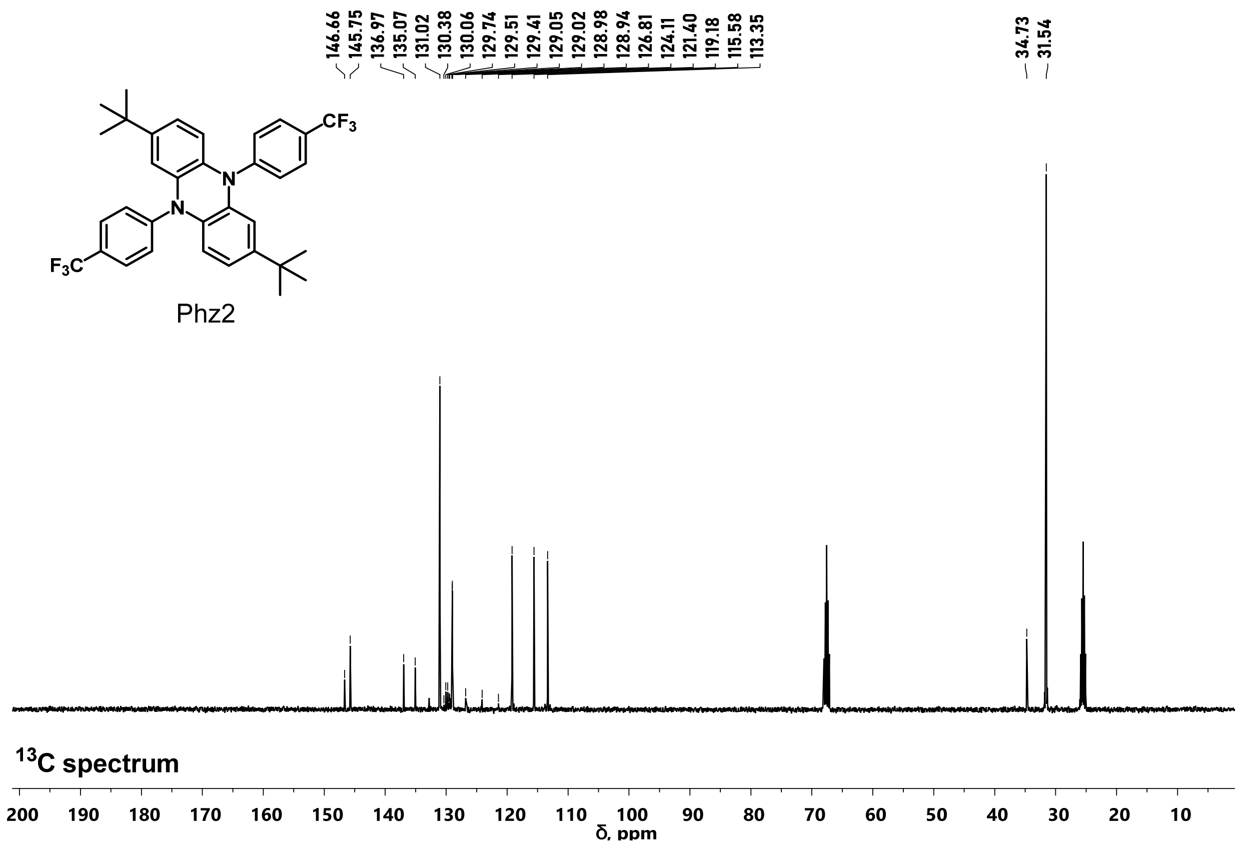
146.66
145.75
136.97
135.07
131.02
130.38
130.06
129.74
129.51
129.41
129.05
129.02
128.98
128.94
126.81
124.11
121.40
119.18
115.58
113.35
34.73
31.54
CF3
N
N
F3C
Phz2
13C spectrum
δ, ppm

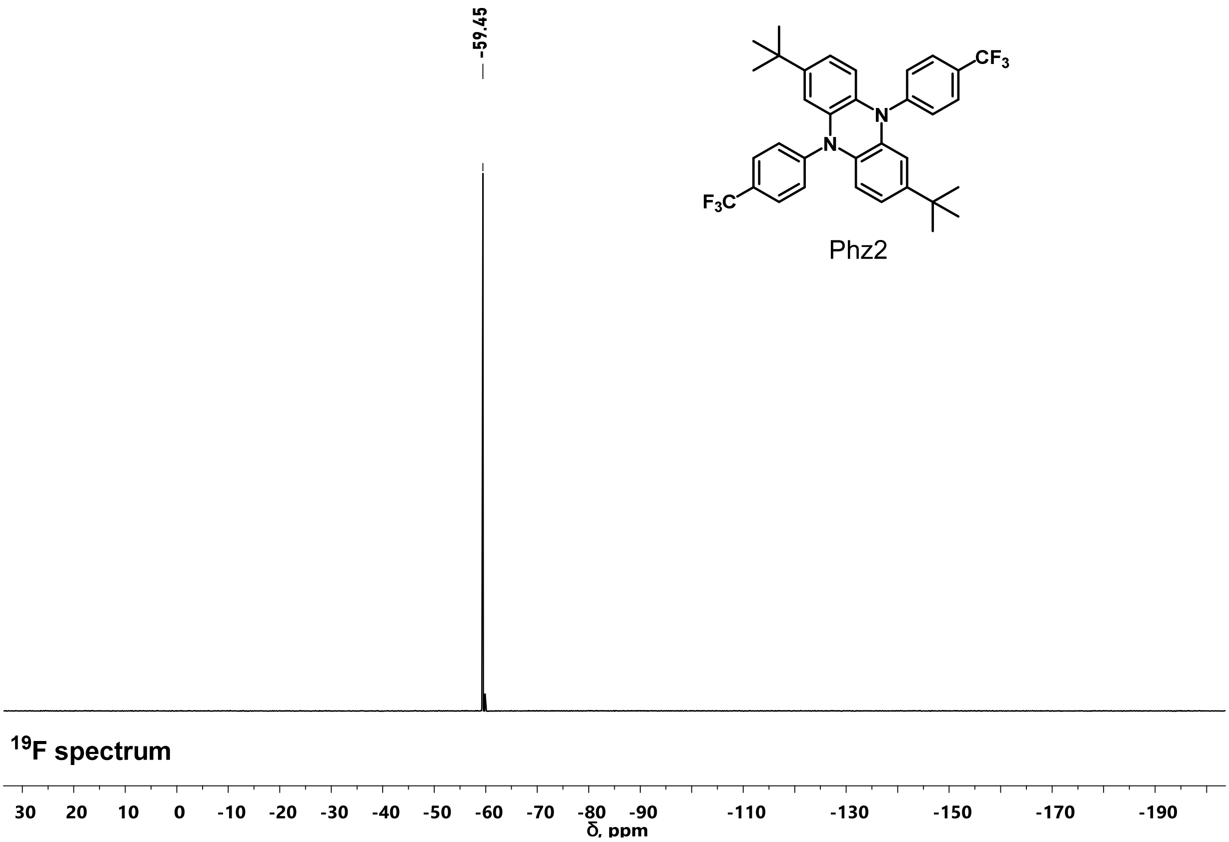

-59.45
CF_3
F_3C
N
Phz2
^{19}F spectrum
30 20 10 0 -10 -20 -30 -40 -50 -60 -70 -80 -90 -110 -130 -150 -170 -190
δ, ppm

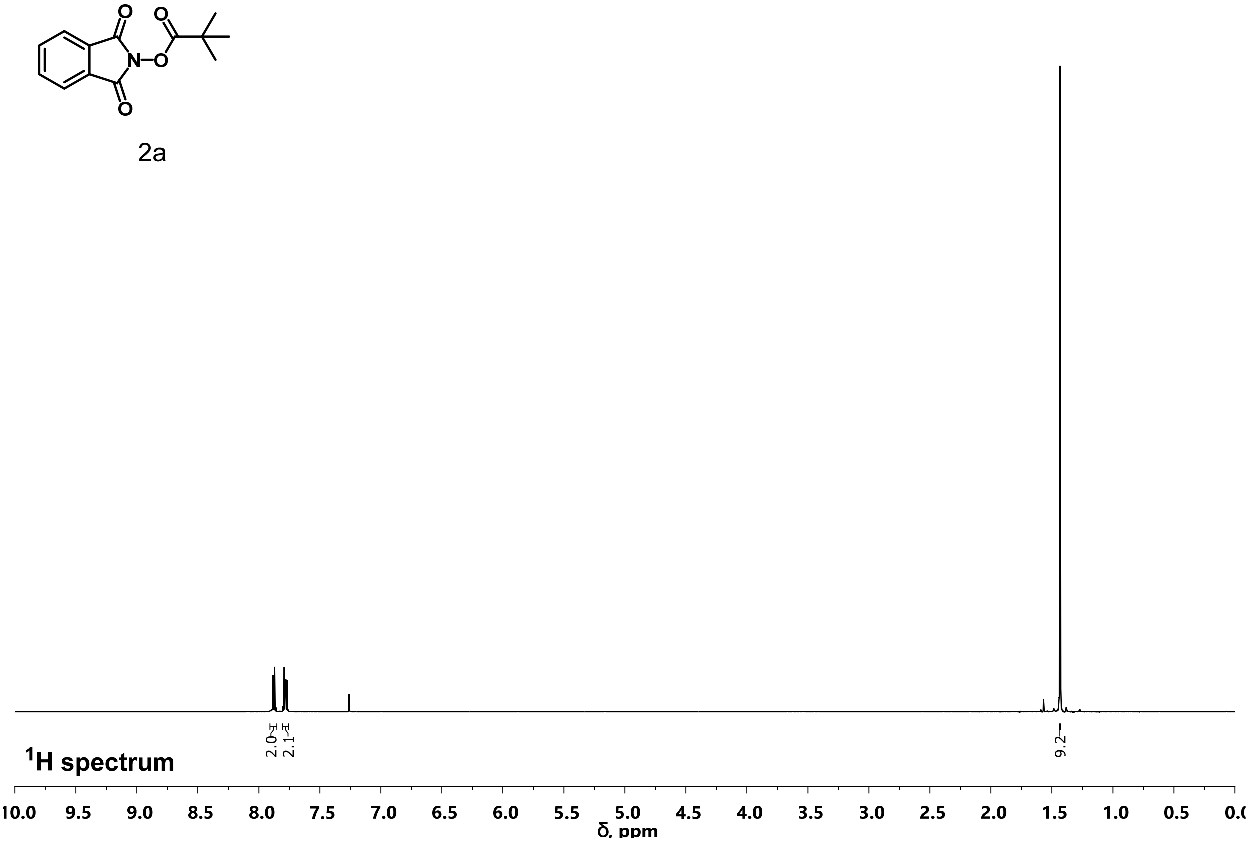

O
N–O
2a
2.0
2.1
9.2
^1H spectrum
10.0 9.5 9.0 8.5 8.0 7.5 7.0 6.5 6.0 5.5 5.0 4.5 4.0 3.5 3.0 2.5 2.0 1.5 1.0 0.5 0.0
δ, ppm

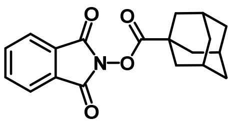

2b

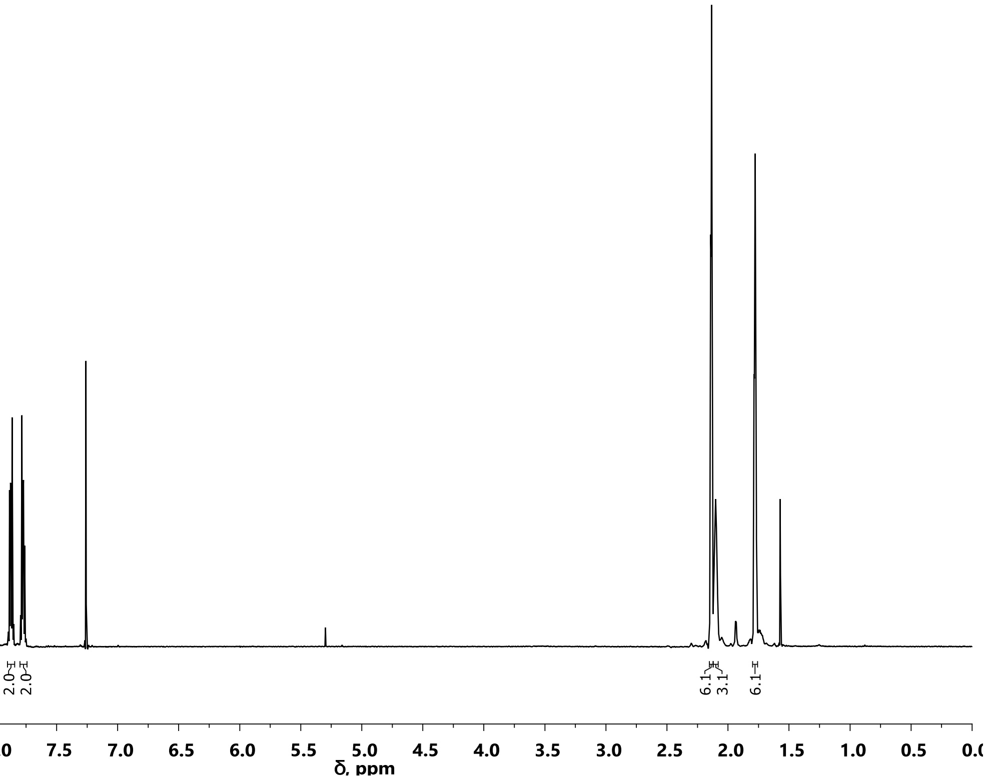

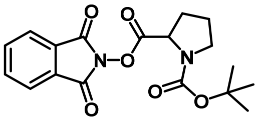

2c

two rotamers

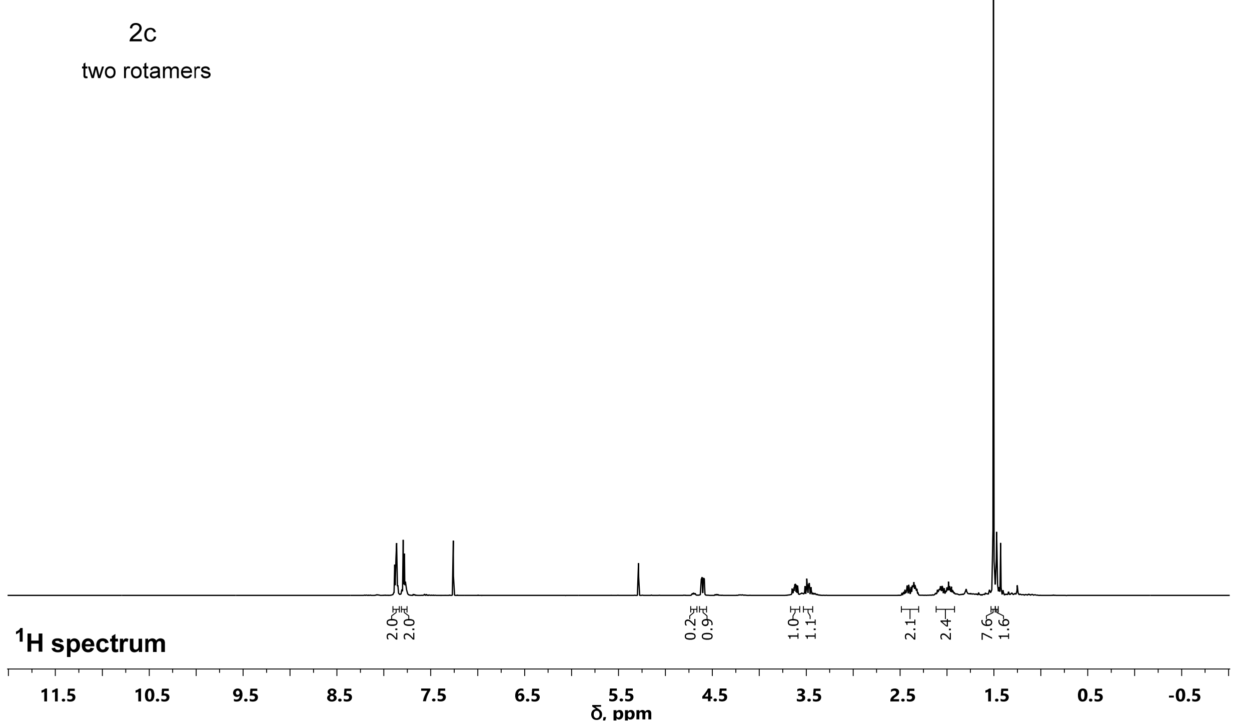

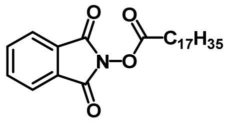
C17H35

2d

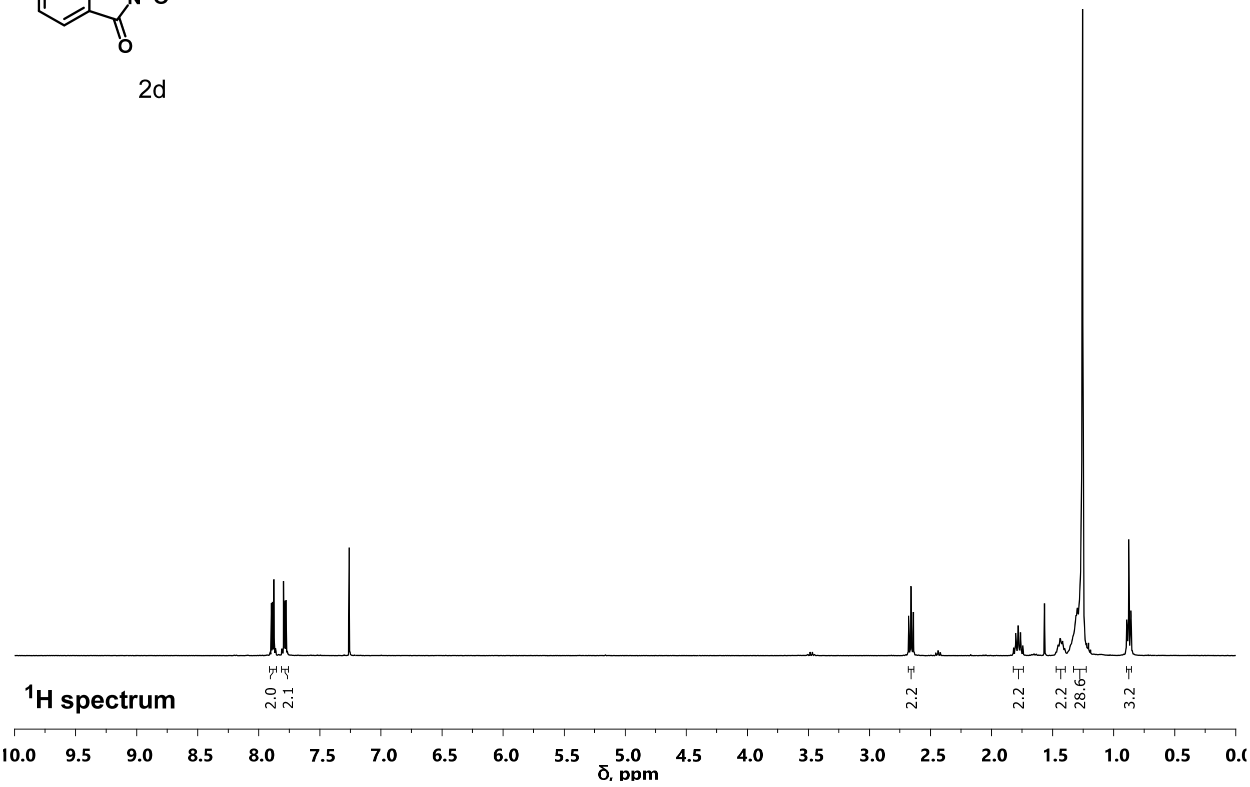
2.0
2.1
2.2
2.2
2.2
28.6
3.2
1H spectrum
10.0 9.5 9.0 8.5 8.0 7.5 7.0 6.5 6.0 5.5 5.0 4.5 4.0 3.5 3.0 2.5 2.0 1.5 1.0 0.5
δ, ppm

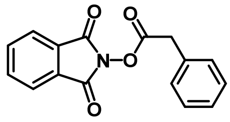

2e

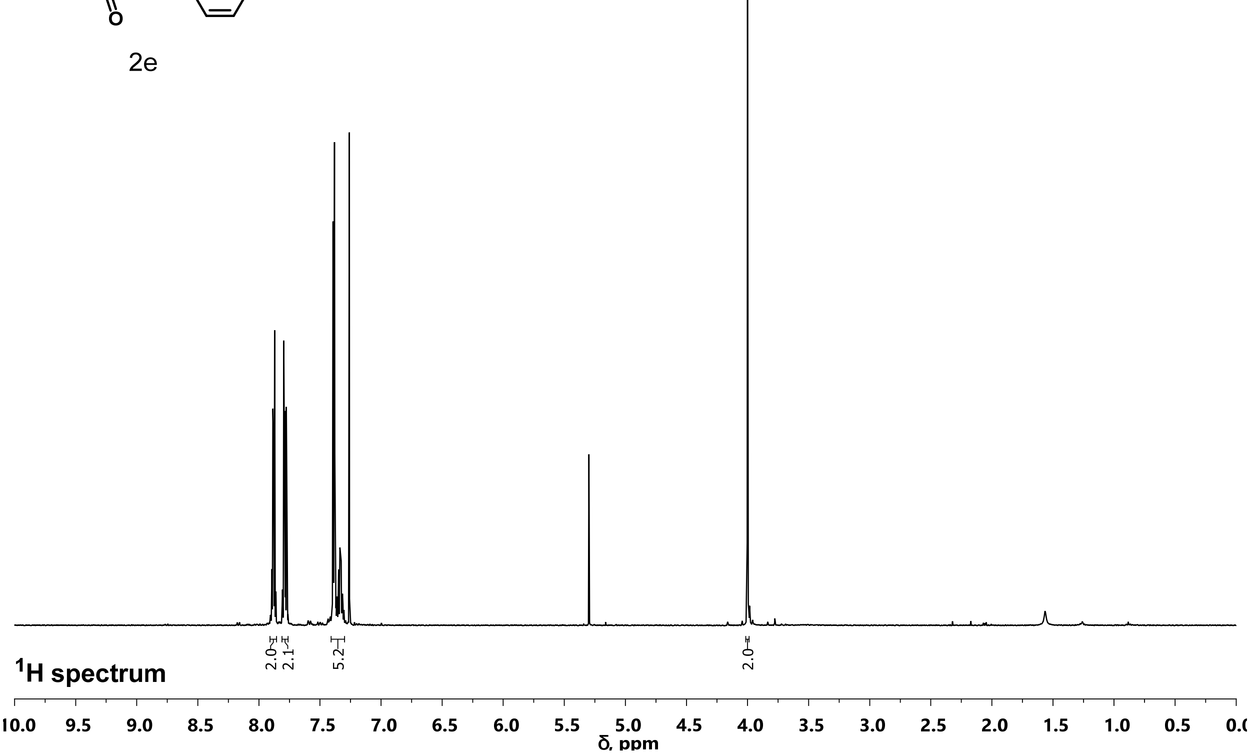
2.0
2.1
5.2
2.0
1H spectrum
10.0 9.5 9.0 8.5 8.0 7.5 7.0 6.5 6.0 5.5 5.0 4.5 4.0 3.5 3.0 2.5 2.0 1.5 1.0 0.5
δ, ppm

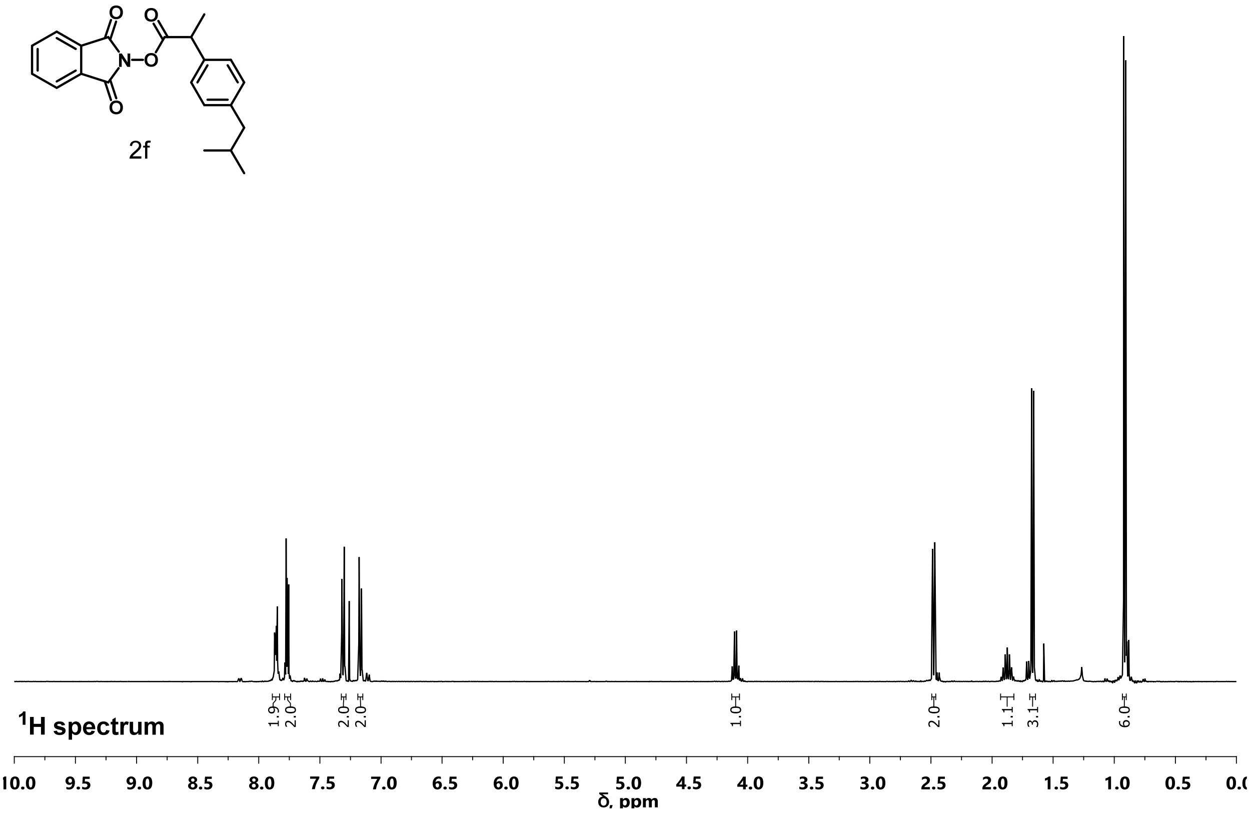
2f
1.9
2.0
2.0
2.0
1.0
2.0
1.1
3.1
6.0
1H spectrum
10.0 9.5 9.0 8.5 8.0 7.5 7.0 6.5 6.0 5.5 5.0 4.5 4.0 3.5 3.0 2.5 2.0 1.5 1.0 0.5 0.0
δ, ppm

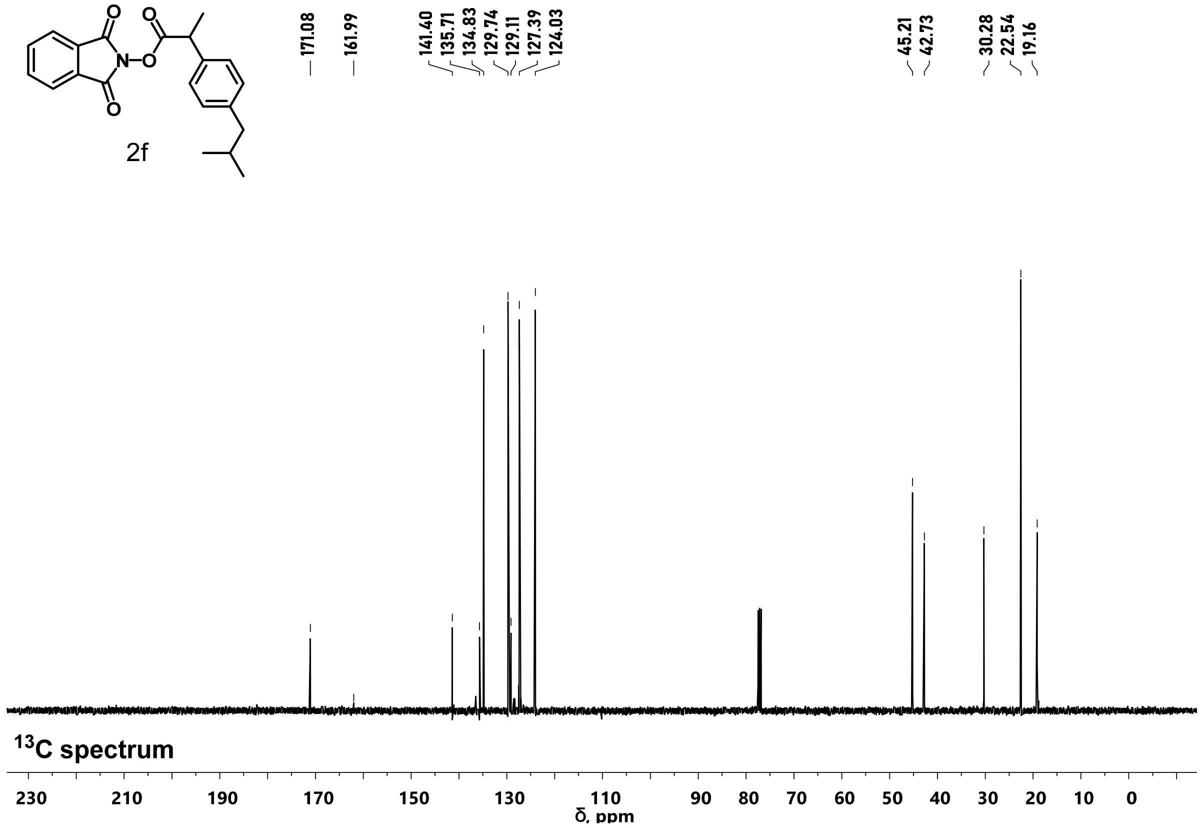
2f
171.08
161.99
141.40
135.71
134.83
129.74
129.11
127.39
124.03
45.21
42.73
30.28
22.54
19.16
13C spectrum
230 210 190 170 150 130 110 90 80 70 60 50 40 30 20 10 0
δ, ppm

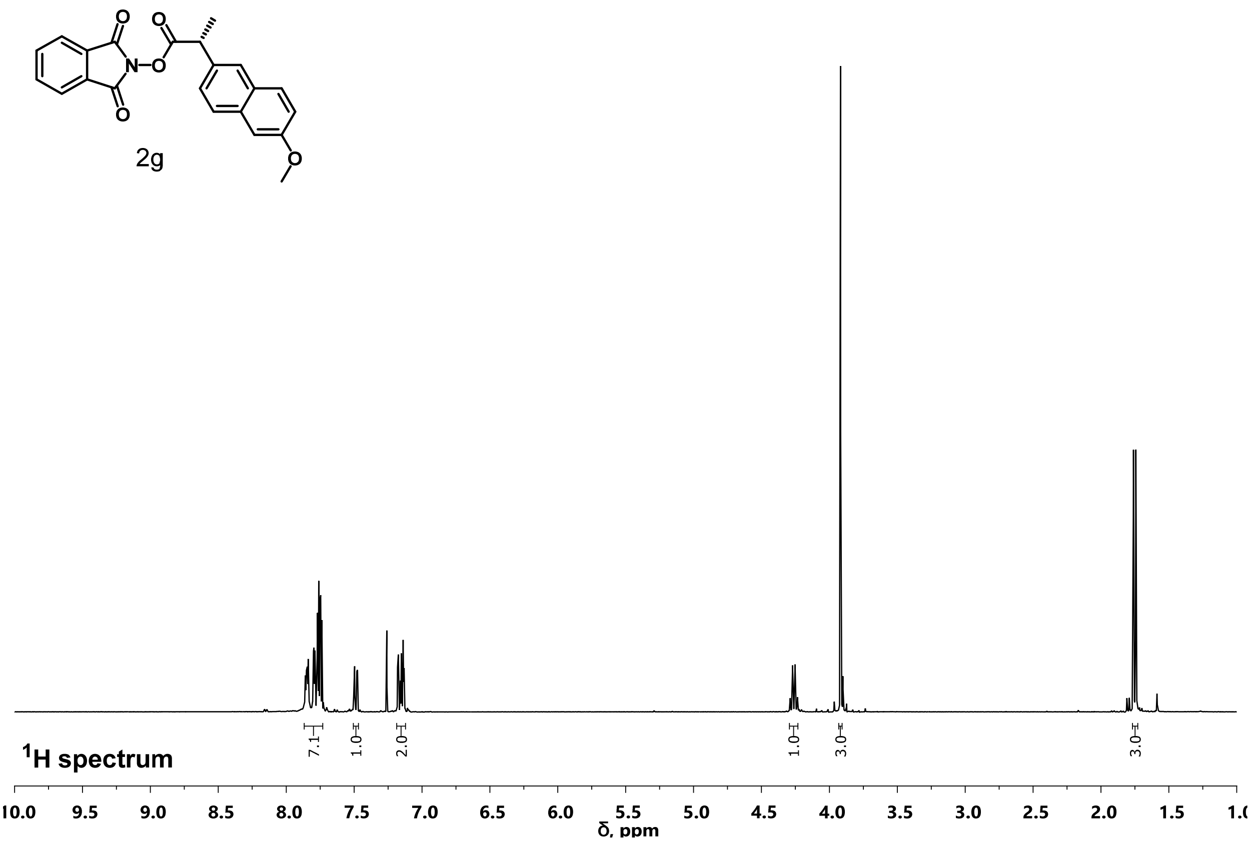
2g
7.1
1.0
2.0
1.0
3.0
3.0
¹H spectrum
10.0 9.5 9.0 8.5 8.0 7.5 7.0 6.5 6.0 5.5 5.0 4.5 4.0 3.5 3.0 2.5 2.0 1.5
δ, ppm

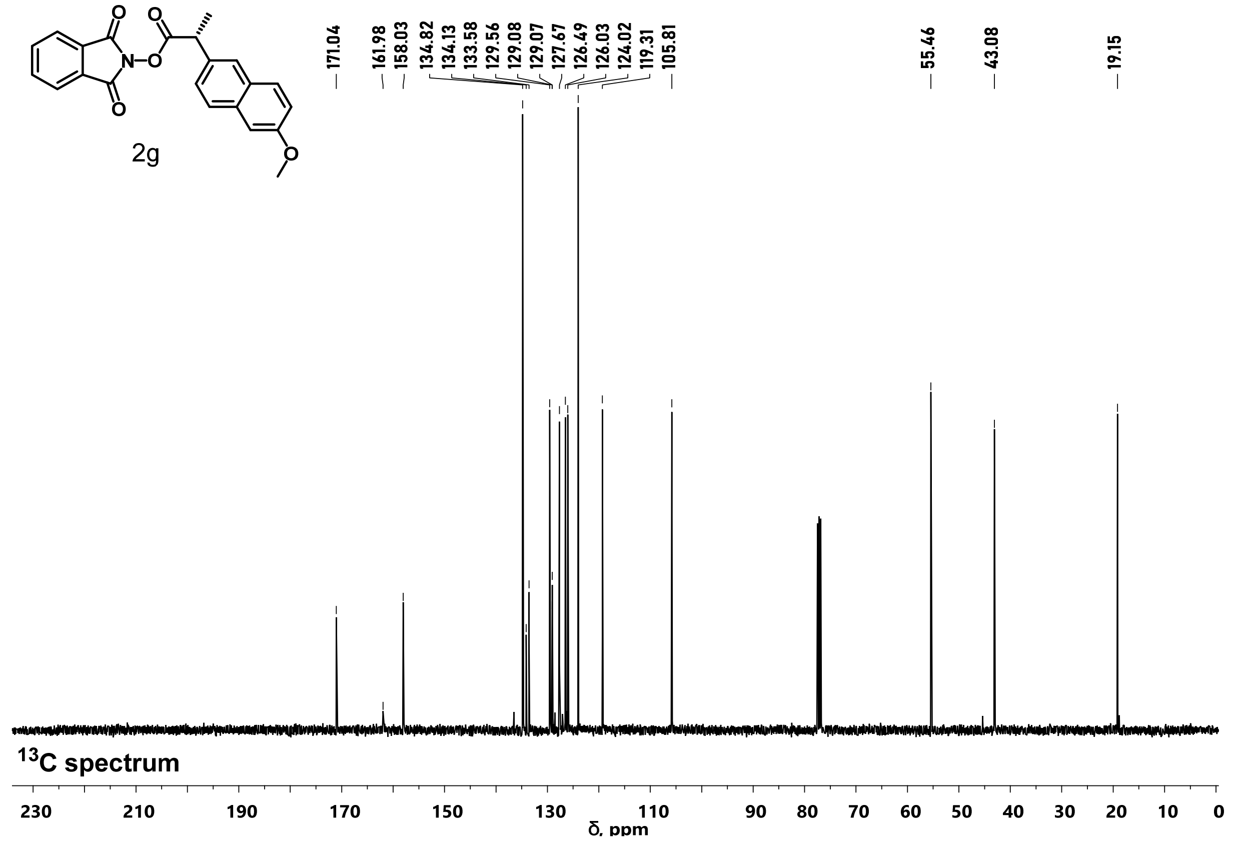
2g
171.04
161.98
158.03
134.82
134.13
133.58
129.56
129.08
129.07
127.67
126.49
126.03
124.02
119.31
105.81
55.46
43.08
19.15
¹³C spectrum
230 210 190 170 150 130 110 90 80 70 60 50 40 30 20 10 0
δ, ppm

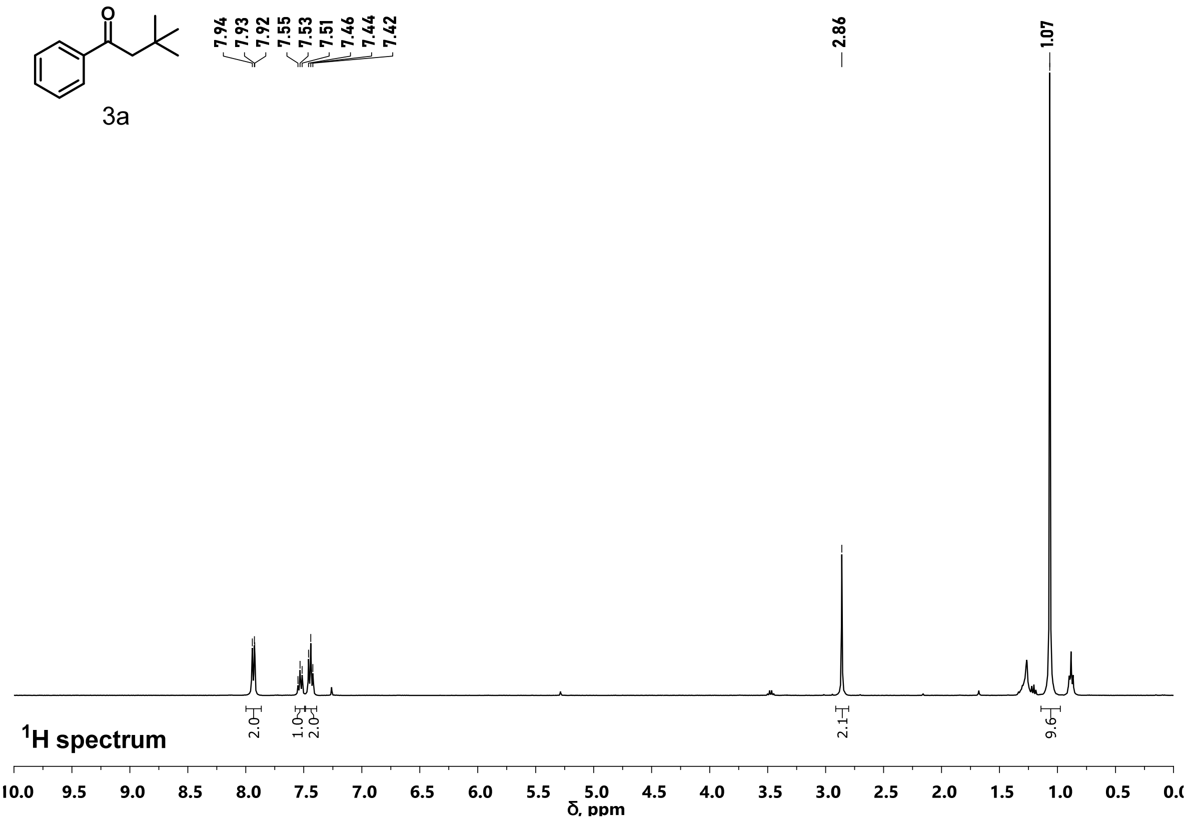
3a
7.94
7.93
7.92
7.55
7.53
7.51
7.46
7.44
7.42
2.86
1.07
2.0
1.0
2.0
2.1
9.6
1H spectrum
10.0 9.5 9.0 8.5 8.0 7.5 7.0 6.5 6.0 5.5 5.0 4.5 4.0 3.5 3.0 2.5 2.0 1.5 1.0 0.5
δ, ppm

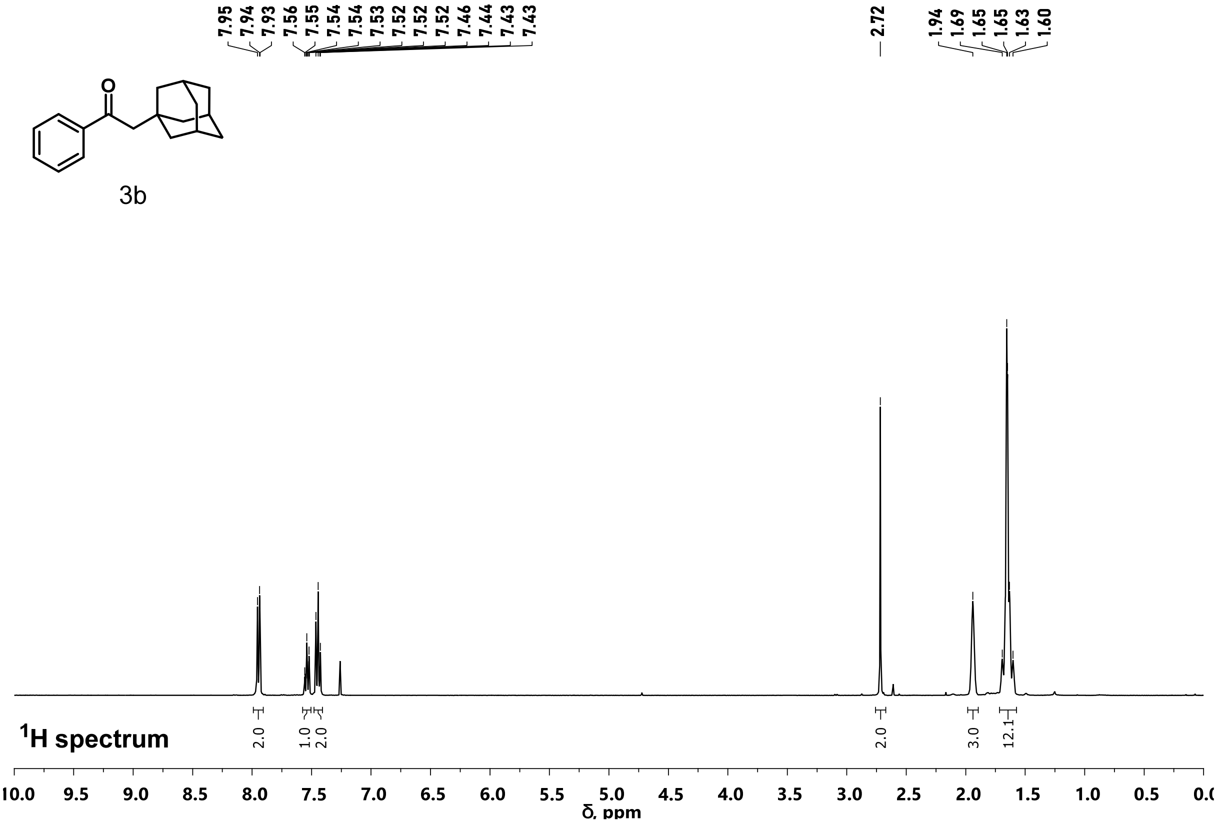
7.95
7.94
7.93
7.56
7.55
7.54
7.53
7.52
7.52
7.52
7.46
7.44
7.43
7.43
2.72
1.94
1.69
1.65
1.65
1.63
1.60
3b
2.0
1.0
2.0
2.0
3.0
12.1
1H spectrum
10.0 9.5 9.0 8.5 8.0 7.5 7.0 6.5 6.0 5.5 5.0 4.5 4.0 3.5 3.0 2.5 2.0 1.5 1.0 0.5
δ, ppm

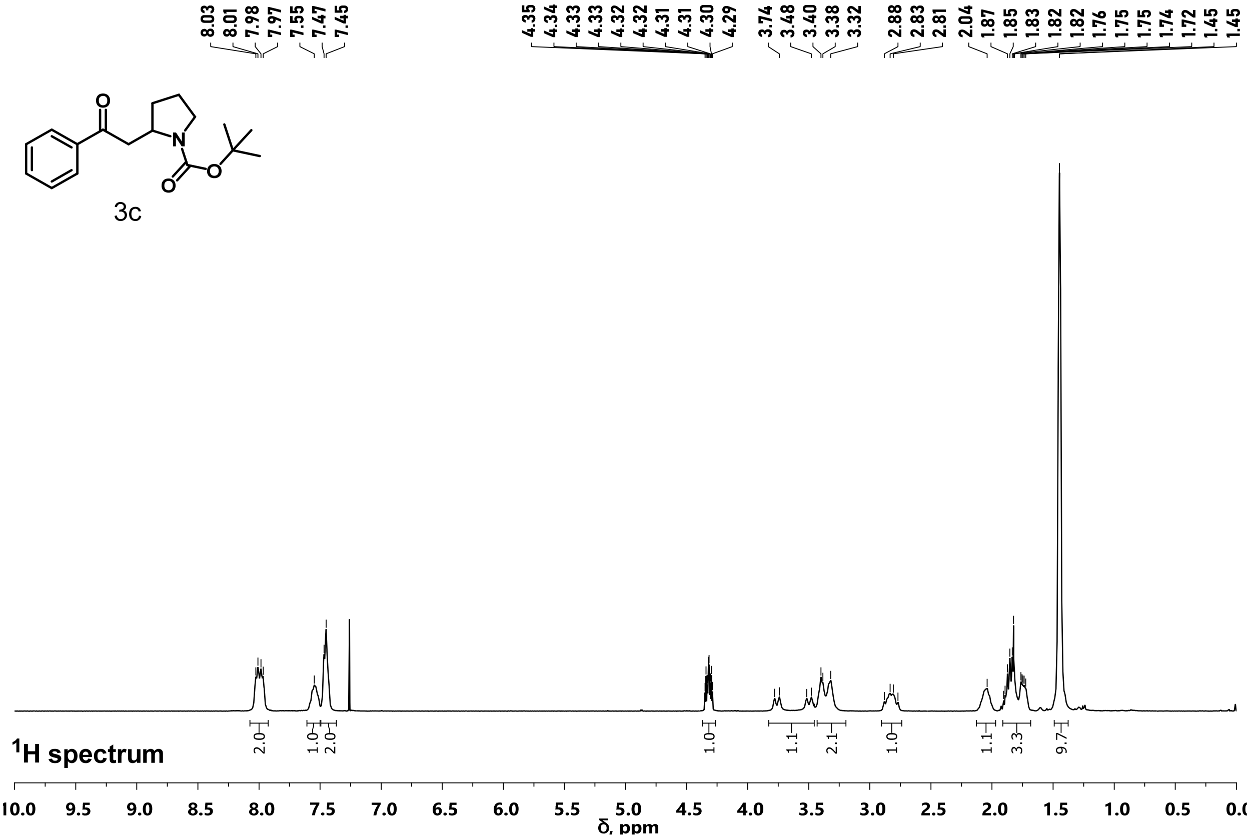
3c
^{1}H spectrum
δ, ppm

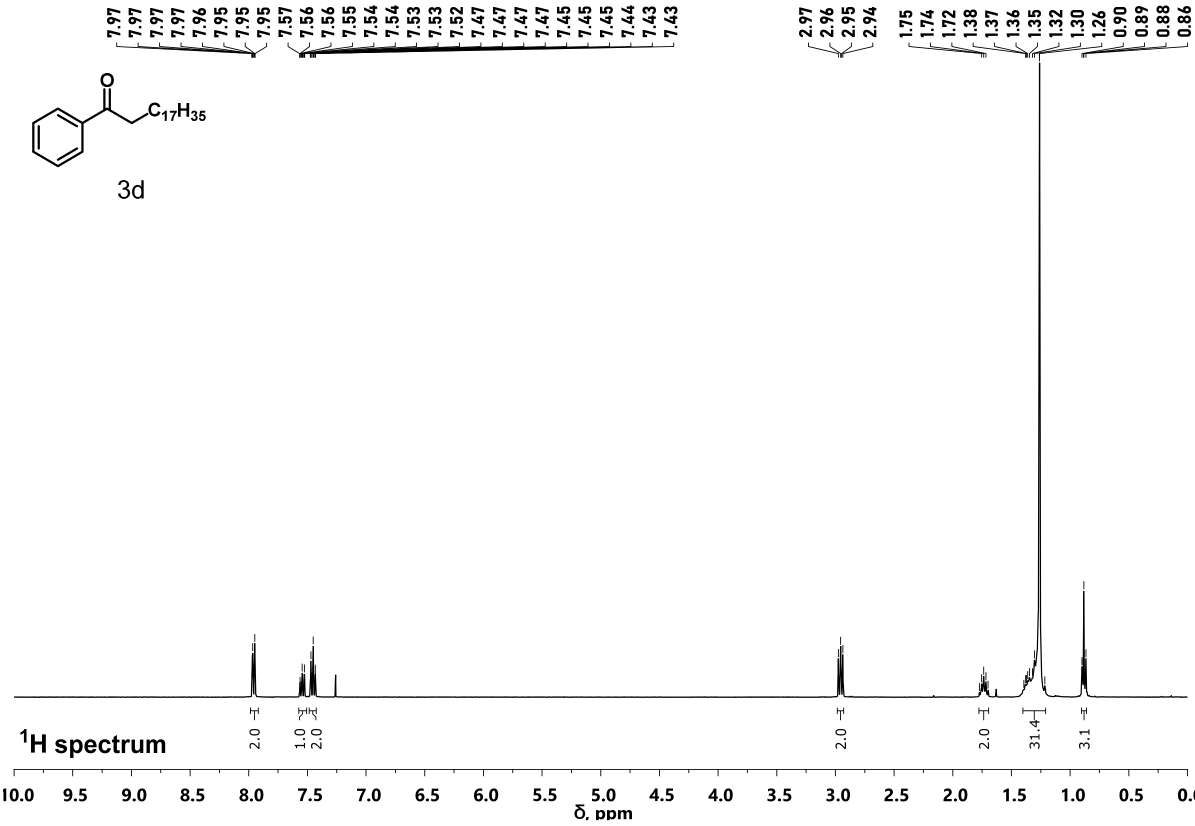
$C_{17}H_{35}$
3d
^{1}H spectrum
δ, ppm

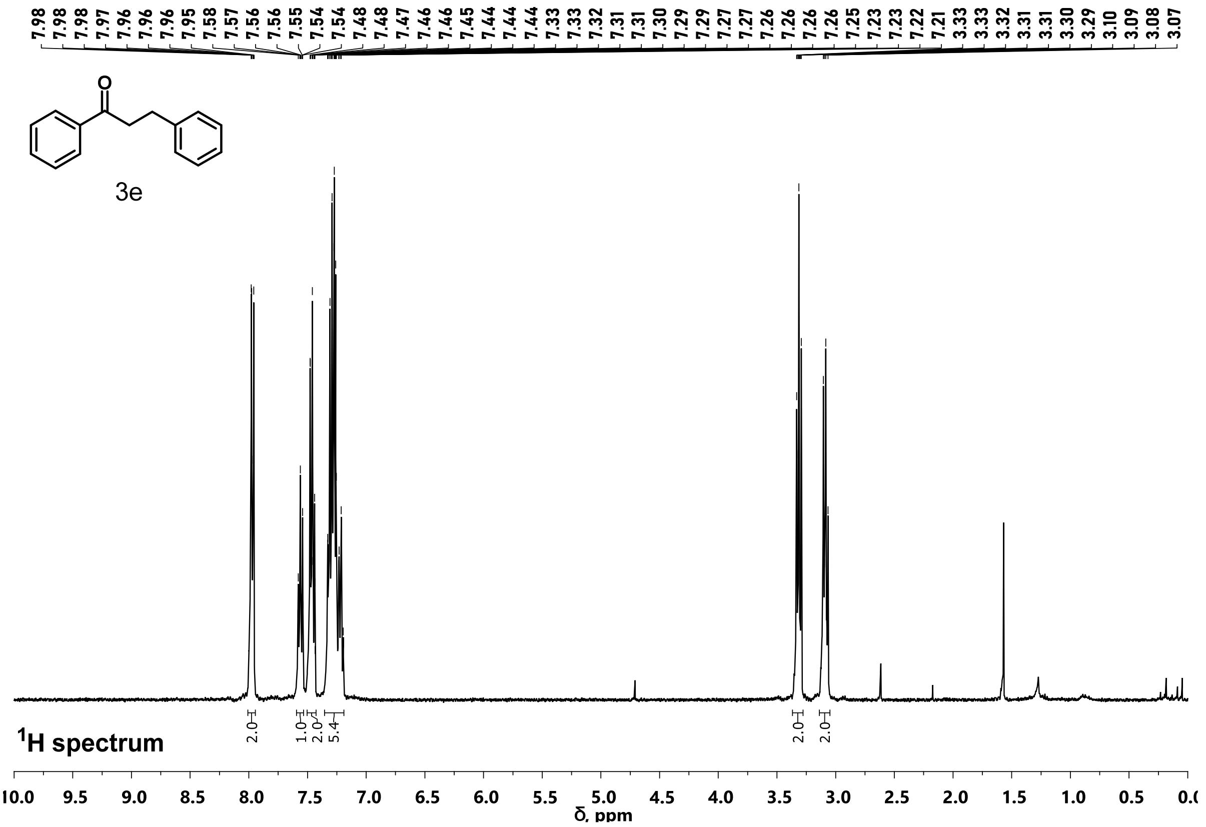
7.98 7.98 7.98 7.97 7.96 7.96 7.96 7.95 7.58 7.57 7.56 7.56 7.55 7.54 7.54 7.48 7.48 7.47 7.46 7.46 7.45 7.44 7.44 7.44 7.33 7.33 7.32 7.31 7.31 7.30 7.29 7.29 7.27 7.27 7.26 7.26 7.26 7.25 7.23 7.23 7.22 7.21 3.33 3.33 3.32 3.31 3.31 3.30 3.29 3.10 3.09 3.08 3.07
O
3e
2.0 1.0 2.0 5.4 2.0 2.0
^{1}H spectrum
10.0 9.5 9.0 8.5 8.0 7.5 7.0 6.5 6.0 5.5 5.0 4.5 4.0 3.5 3.0 2.5 2.0 1.5 1.0 0.5 0.0
δ, ppm

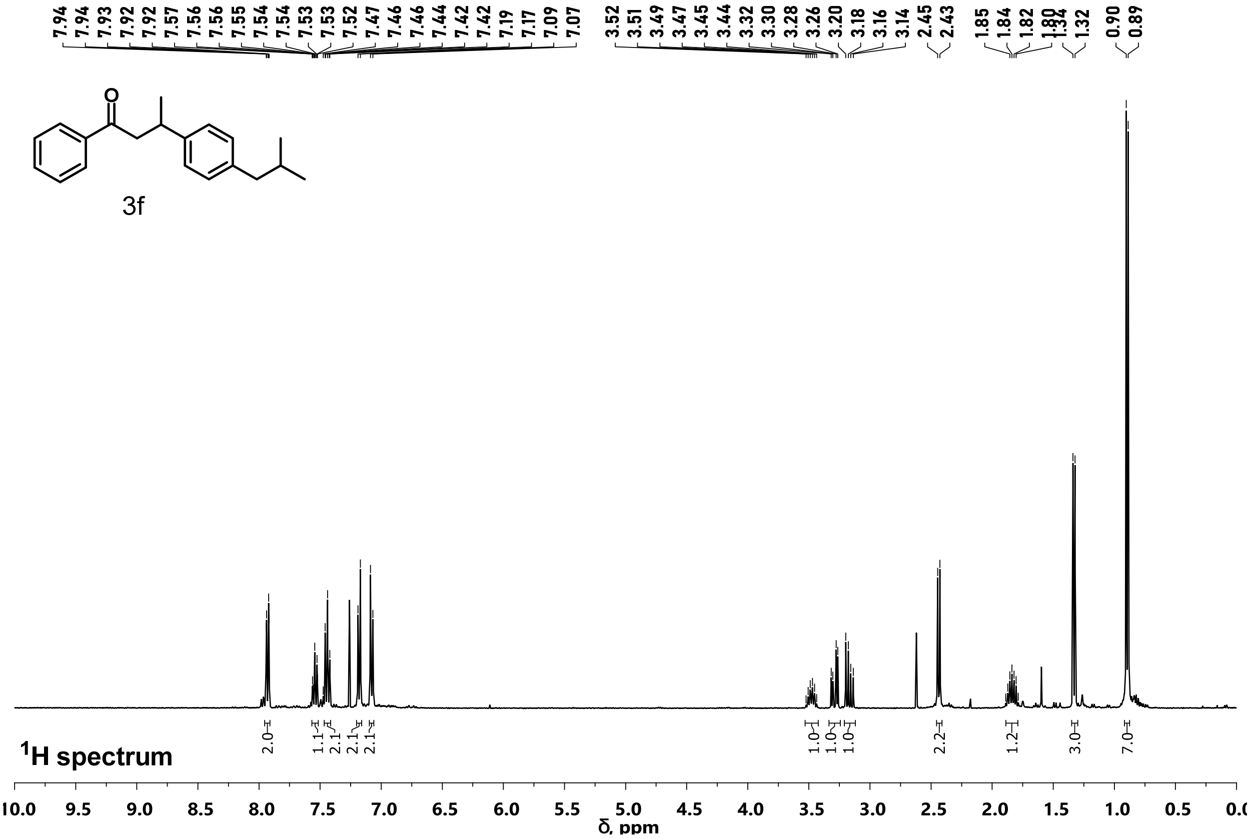
7.94 7.94 7.93 7.92 7.92 7.57 7.56 7.56 7.55 7.54 7.54 7.53 7.53 7.52 7.47 7.46 7.46 7.44 7.42 7.42 7.19 7.17 7.09 7.07 3.52 3.51 3.49 3.47 3.45 3.44 3.32 3.30 3.28 3.26 3.20 3.18 3.16 3.14 2.45 2.43 1.85 1.84 1.82 1.80 1.34 1.32 0.90 0.89
O
3f
2.0 1.1 2.1 2.1 2.1 1.0 1.0 1.0 2.2 1.2 3.0 7.0
^{1}H spectrum
10.0 9.5 9.0 8.5 8.0 7.5 7.0 6.5 6.0 5.5 5.0 4.5 4.0 3.5 3.0 2.5 2.0 1.5 1.0 0.5 0.0
δ, ppm

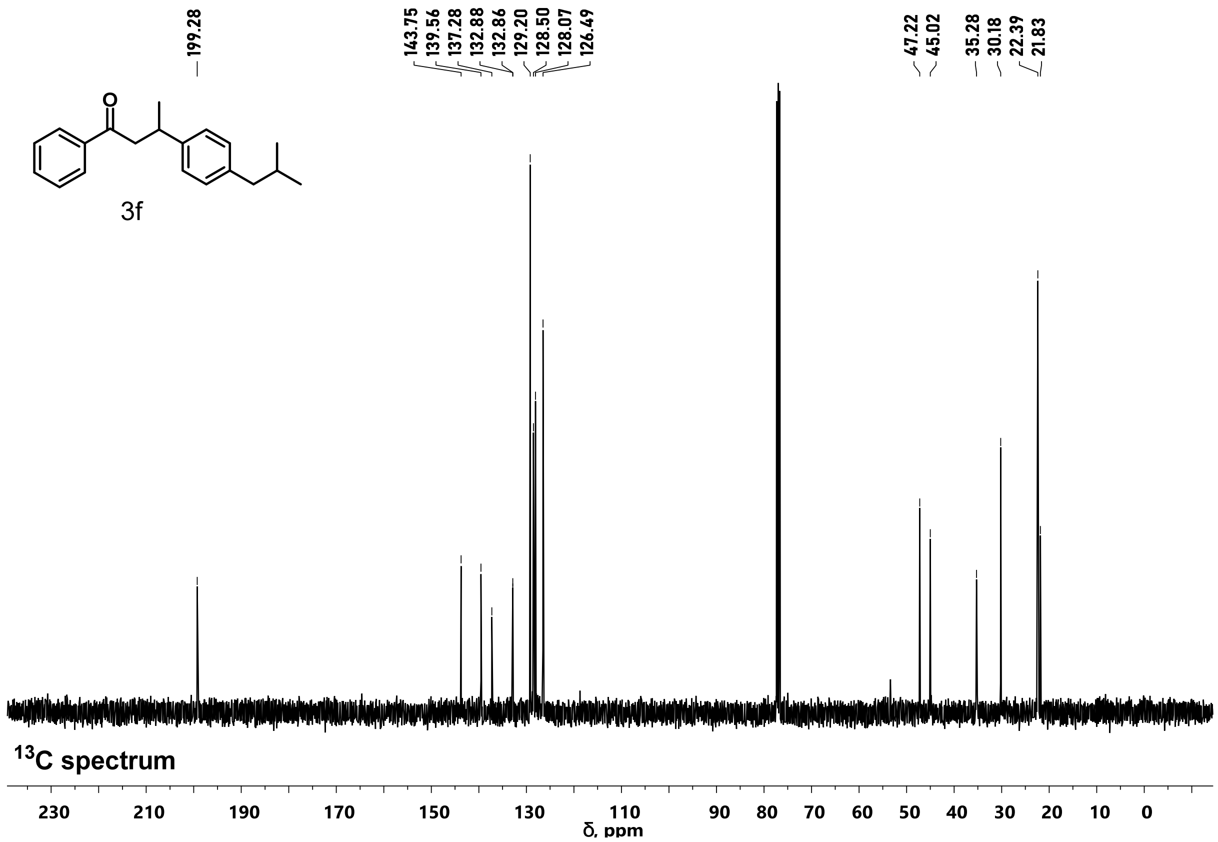

3f

^{13}C spectrum

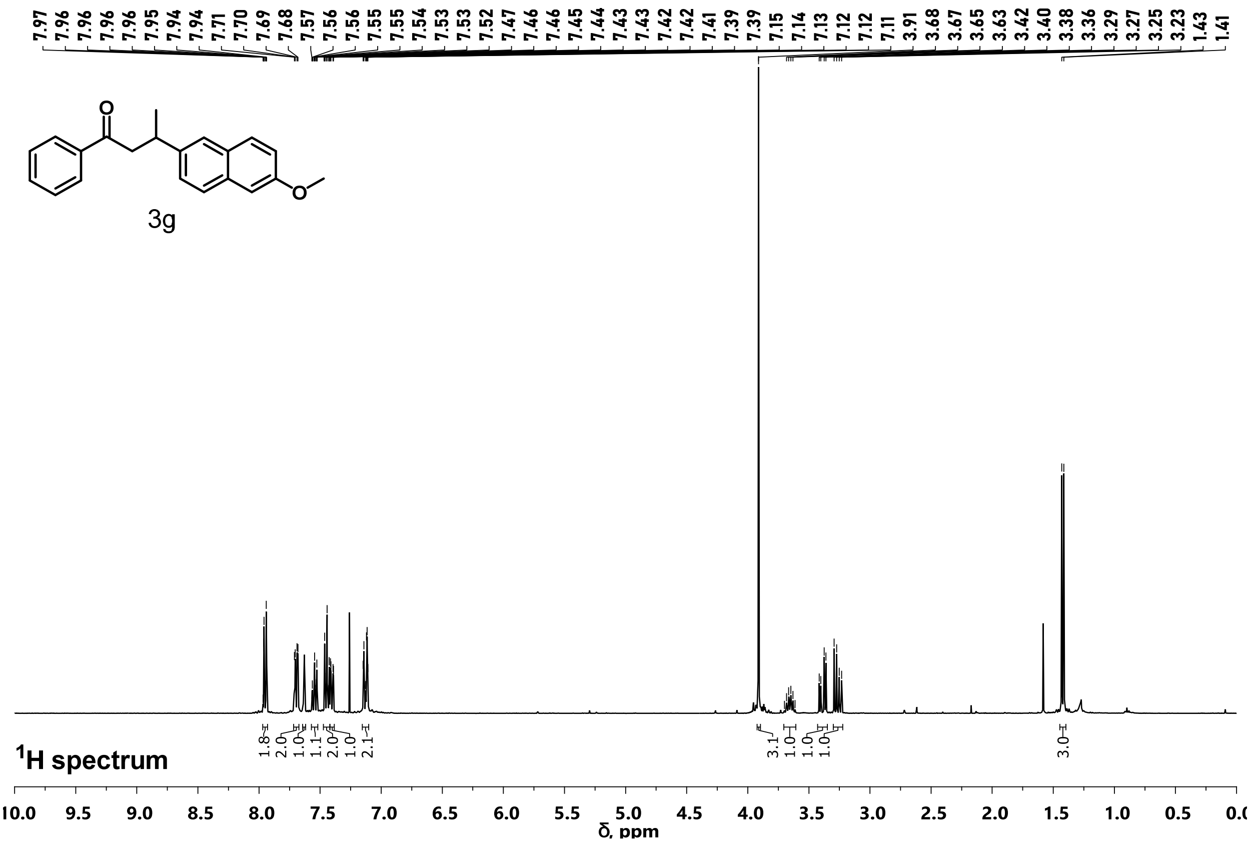

3g

^{1}H spectrum

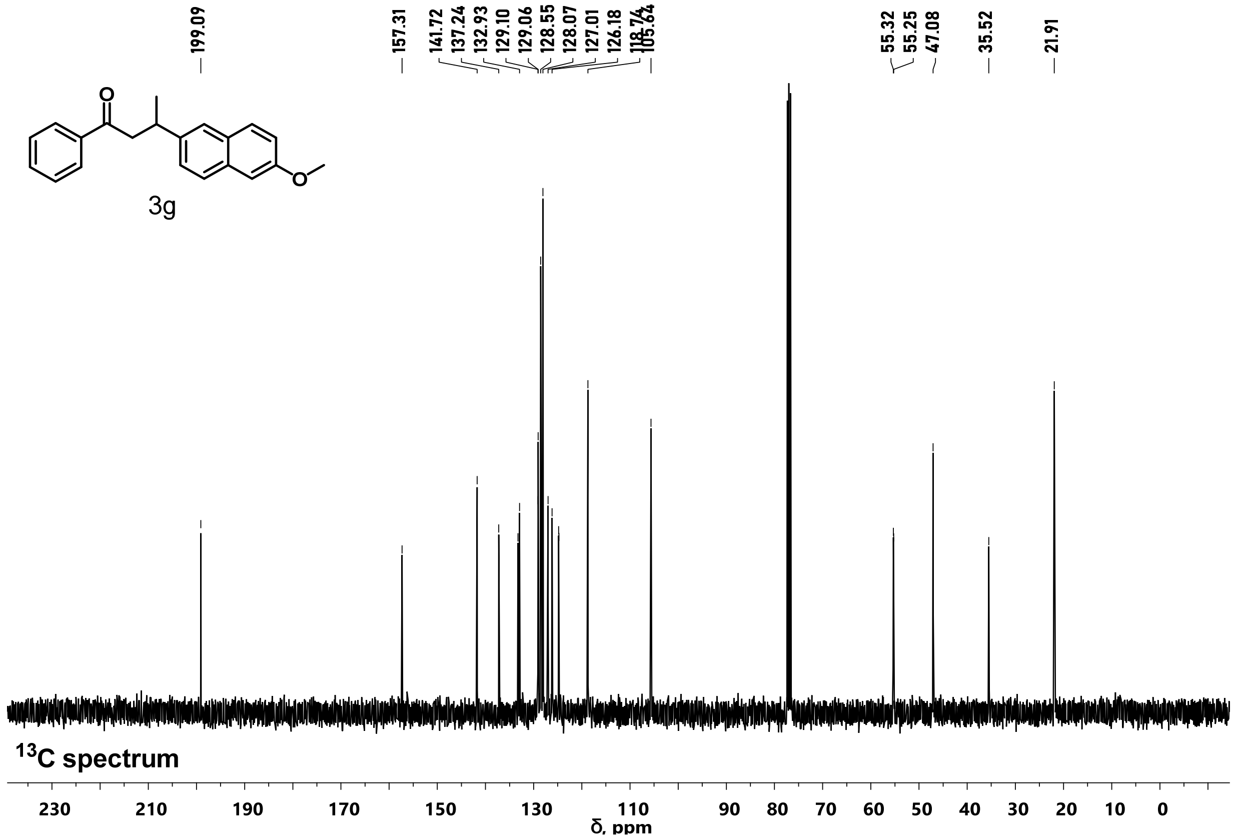
199.09
157.31
141.72
137.24
132.93
129.10
129.06
128.55
128.07
127.01
126.18
118.74
105.64
55.32
55.25
47.08
35.52
21.91
O
O
3g
13C spectrum
230
210
190
170
150
130
110
90
80
70
60
50
40
30
20
10
0
δ, ppm

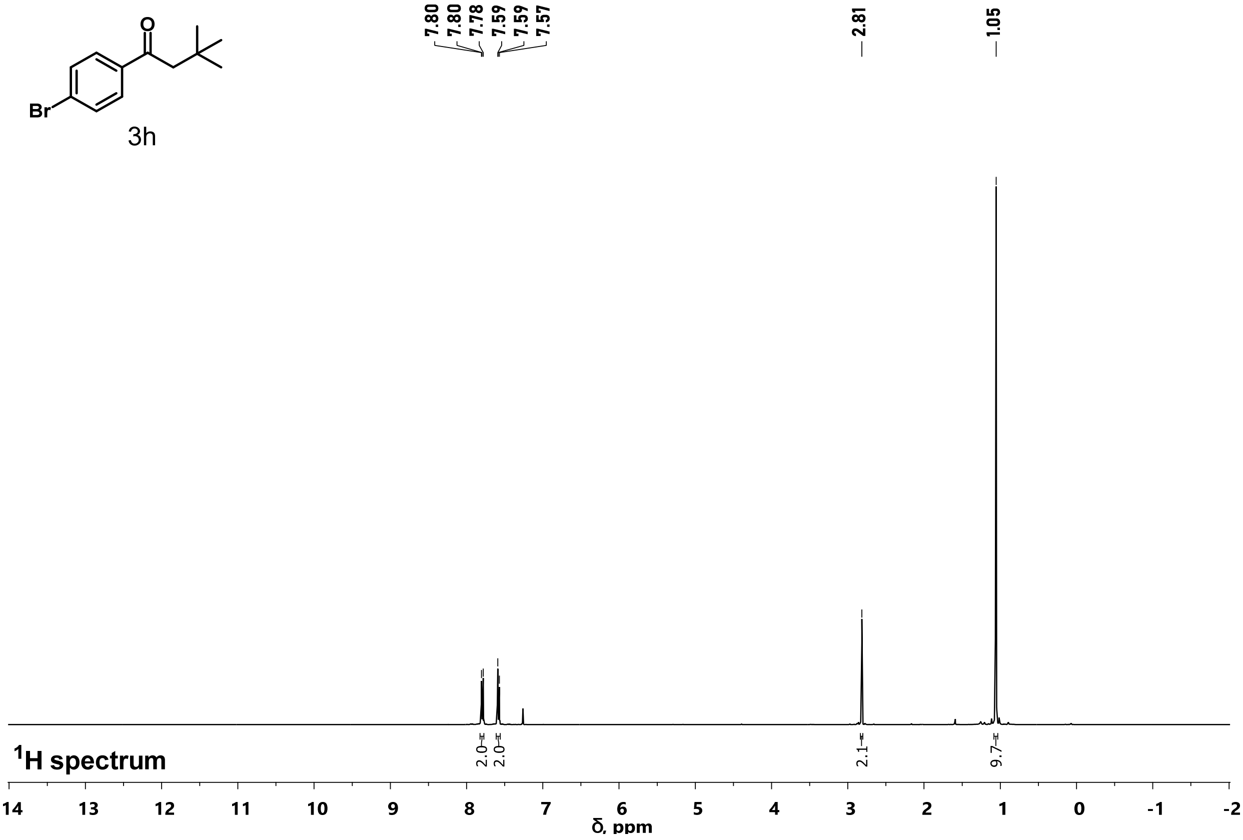
7.80
7.80
7.78
7.59
7.59
7.57
2.81
1.05
O
Br
3h
2.0
2.0
2.1
9.7
1H spectrum
14
13
12
11
10
9
8
7
6
5
4
3
2
1
0
-1
-2
δ, ppm
